# Molecular mechanisms and consequences of TDP-43 phosphorylation in neurodegeneration

**DOI:** 10.1186/s13024-025-00839-8

**Published:** 2025-05-08

**Authors:** Elise A. Kellett, Adekunle T. Bademosi, Adam K. Walker

**Affiliations:** 1 Neurodegeneration Pathobiology Laboratory, Clem Jones Centre for Ageing Dementia Research, Queensland Brain Institute, The University of Queensland, St Lucia, 4072 QLD Australia; 2 Sydney Pharmacy School, Faculty of Medicine and Health, The University of Sydney, Camperdown, 2006 NSW Australia; 3 Charles Perkins Centre, The University of Sydney, Camperdown, 2006 NSW Australia

**Keywords:** TDP-43, Phosphorylation, Amyotrophic lateral sclerosis, Frontotemporal dementia, Post-translational modifications, Neurodegeneration, Kinase, Phosphatase

## Abstract

Increased phosphorylation of TDP-43 is a pathological hallmark of several neurodegenerative disorders, including amyotrophic lateral sclerosis (ALS) and frontotemporal dementia (FTD). However, the regulation and roles of TDP-43 phosphorylation remain incompletely understood. A variety of techniques have been utilized to understand TDP-43 phosphorylation, including kinase/phosphatase manipulation, phosphomimic variants, and genetic, physical, or chemical inducement in a variety of cell cultures and animal models, and via analyses of post-mortem human tissues. These studies have produced conflicting results: suggesting incongruously that TDP-43 phosphorylation may either drive disease progression or serve a neuroprotective role. In this review, we explore the roles of regulators of TDP-43 phosphorylation including the putative TDP-43 kinases c-Abl, CDC7, CK1, CK2, IKKβ, p38α/MAPK14, MEK1, TTBK1, and TTBK2, and TDP-43 phosphatases PP1, PP2A, and PP2B, in disease. Building on recent studies, we also examine the consequences of TDP-43 phosphorylation on TDP-43 pathology, especially related to TDP-43 mislocalisation, liquid–liquid phase separation, aggregation, and neurotoxicity. By comparing conflicting findings from various techniques and models, this review highlights both the discrepancies and unresolved aspects in the understanding of TDP-43 phosphorylation. We propose that the role of TDP-43 phosphorylation is site and context dependent, and includes regulation of liquid–liquid phase separation, subcellular mislocalisation, and degradation. We further suggest that greater consideration of the normal functions of the regulators of TDP-43 phosphorylation that may be perturbed in disease is warranted. This synthesis aims to build towards a comprehensive understanding of the complex role of TDP-43 phosphorylation in the pathogenesis of neurodegeneration.

**Graphical Abstract:**

TDP-43 is subject to phosphorylation by kinases and dephosphorylation by phosphatases, which variably impacts protein localisation, aggregation, and neurotoxicity in neurodegenerative diseases.

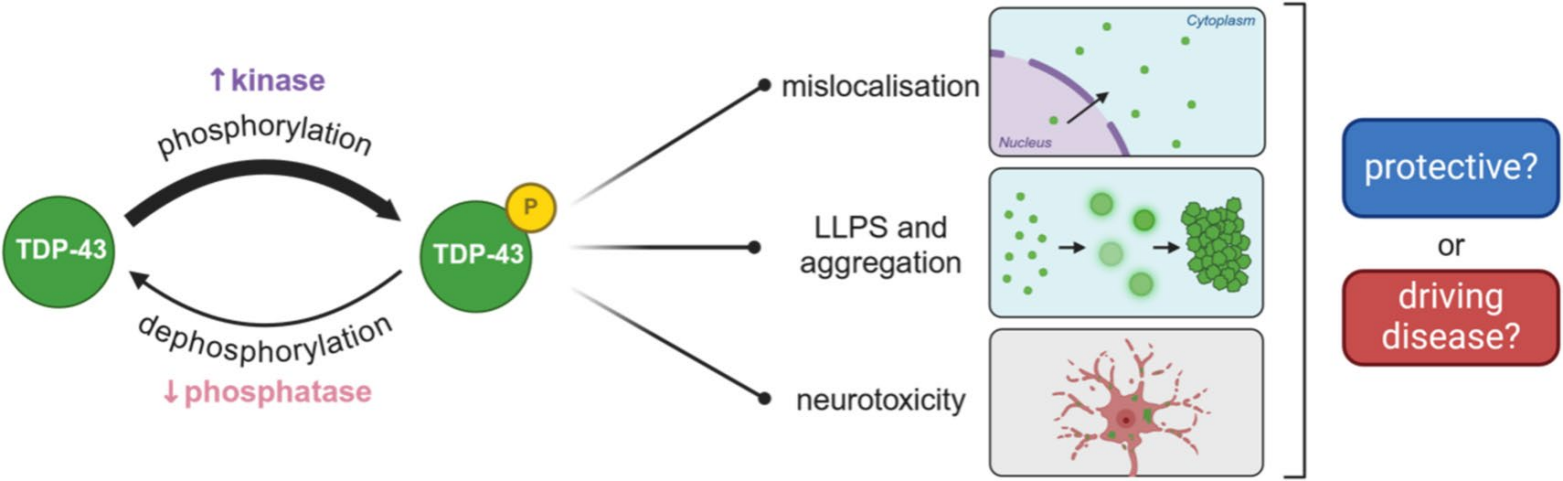

## Background

Amyotrophic lateral sclerosis (ALS), frontotemporal dementia (FTD), and limbic-predominant age-related TDP-43 encephalopathy (LATE) are fatal and devastating neurodegenerative disorders characterised by the cytoplasmic mislocalisation and aggregation of RNA-binding proteins. Among these proteins, TAR DNA-binding protein 43 (TDP-43) is the primary aggregating protein driving pathology in approximately 97% of ALS, 50% of FTD, and all LATE cases [1, 2]. Furthermore, TDP-43-positive aggregates have been observed in a subset of other neurodegenerative disorders including Alzheimer’s disease, Parkinson’s disease, and Huntington’s disease (HD) [3–10]. As a unifying feature among the heterogeneity between and within these disorders, TDP-43 aggregation and abnormal TDP-43 post-translation modifications (PTMs), particularly phosphorylation, has emerged as a key pathological hallmarks of TDP-43 proteinopathy. Despite this, the causes, implications, and contributions of aberrant TDP-43 phosphorylation in disease pathogenesis remain unclear [11–13]. While TDP-43 phosphorylation correlated with disease progression [14–30], emerging evidence suggests that TDP-43 phosphorylation may be part of a neuro-protective mechanism [31–35]. This review discusses the regulators, timing, and roles of TDP-43 phosphorylation to explore whether it is protective or disease-contributing.

### TDP-43

#### Function

TDP-43 is an essential RNA and DNA binding protein encoded by the human *TARDBP* gene, with vital roles in gene expression and RNA metabolism. This includes critical processes such as transcription, translation, RNA splicing, and mRNA stability (reviewed in [36]). TDP-43 interacts with over 4,000 mRNA transcripts, with high specificity towards UG-rich RNA sequences, and self-regulates expression levels through a negative feedback loop by destabilising its own mRNA [37–39]. TDP-43 plays an important role in RNA splicing, with loss of nuclear TDP-43 causing mis-splicing with emerging implications for neurodegenerative pathology. For example, TDP-43 mislocalisation induces cryptic exon inclusion in genes that regulate neuronal function such as *UNC13A* and *STMN2*, leading to decreased expression of their translated proteins [40–44]. TDP-43 also plays a key role in cellular stress responses by regulating mRNA levels, such as *G3BP1* [45], to regulate stress granule assembly, apoptosis, axonal transport, and ribonucleoprotein transport [3, 36, 46, 47]. By recruitment to cytoplasmic stress granules in response to cellular stress, TDP-43 also supports stress granule formation and stalling ribosomes [36, 47–49]. TDP-43 is modified by many different PTMs, including acetylation, SUMOylation, ubiquitination, nitrosylation, methylation, C-terminal fragmentation, disulfide bridge formation, citrullination, and phosphorylation [11–13, 50–57]. These modifications likely play crucial roles in regulating TDP-43 function and aggregation propensity [36].

#### Localisation

TDP-43 is a ubiquitously expressed protein translated in the cytoplasm and transported to the nucleus due to its nuclear localisation signal (NLS). Physiological TDP-43 is primarily localised in the nucleus yet can shuttle between the cytoplasm and nucleus [58]. In *Drosophila* neurons, TDP-43 forms cytoplasmic mRNP granules that utilize microtubule-dependent transport to deliver target mRNA to distant neuronal compartments [59]. Similarly, in mouse primary hippocampal neurons, TDP-43 associates with mRNP granules and regulates their transport in dendrites to coordinate mRNA localisation and translation [60]. During cellular stress, TDP-43 translocates to the cytoplasm for stress granules assembly [47, 48]. TDP-43 export to the cytoplasm is a passive process, while being actively transported into the nucleus by importins, for example the importin α/β heterodimer, which recognises the NLS of TDP-43 [61, 62]. TDP-43 is also present at low levels inside mitochondria of human motor and cortical neurons, though these levels are increased in post-mortem ALS/FTLD-TDP spinal cord and frontal context tissue, as well as mice and primary rat motor neurons expressing pathological TDP-43 variants [63–65]. This dynamic localisation underscores the role of TDP-43 in responding to cellular conditions and stress.

#### Structure

**Fig. 1:**
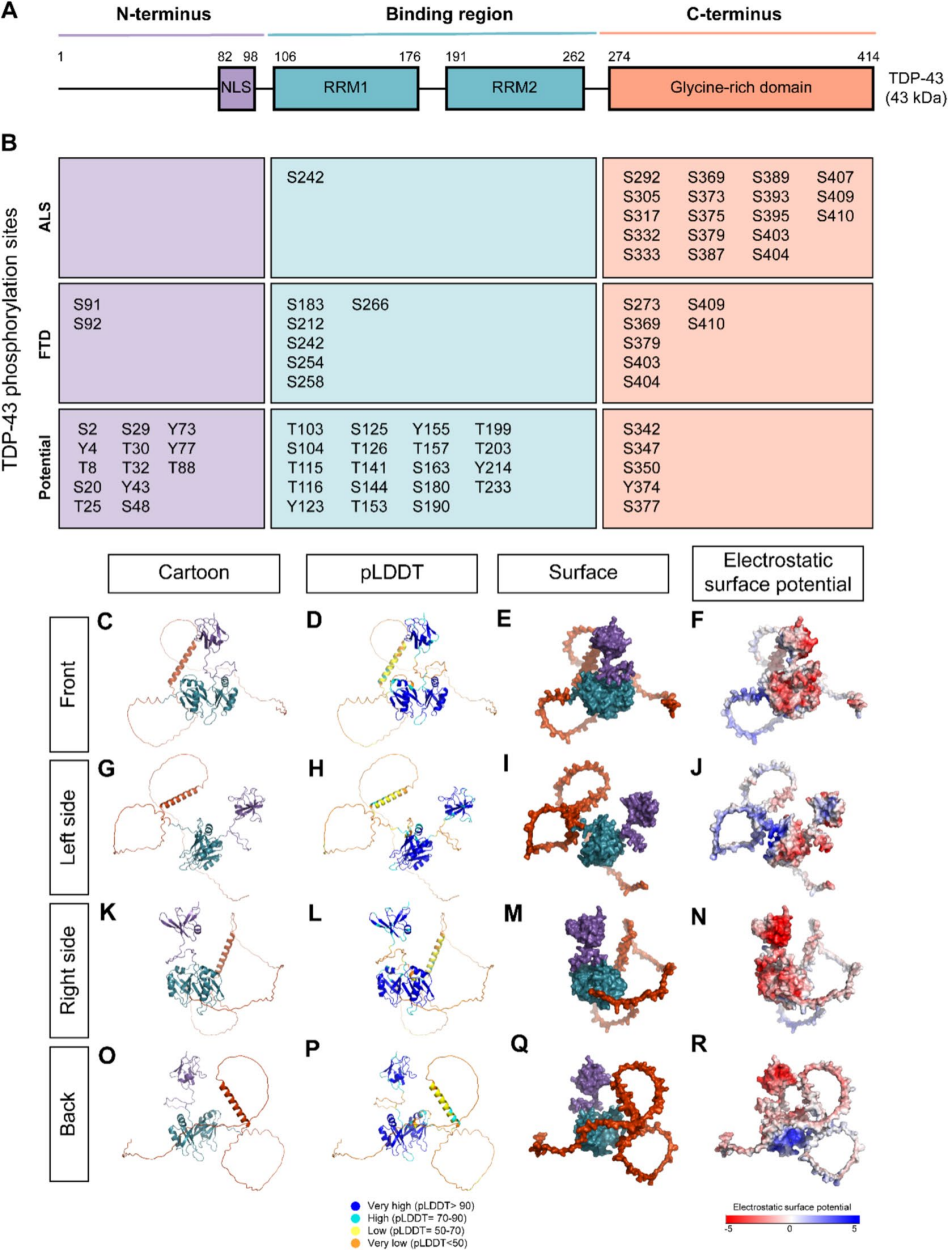
TDP-43 structure and phosphorylation sites. **A** Schematic of TDP-43, showing the N-terminus region (purple) with a nuclear localising signal (NLS), the binding region (blue) with two RNA-recognition motifs (RRM1, RRM2), and the C-terminus region (orange) with a glycine-rich domain. **B** TDP-43 phosphorylation sites detected in post-mortem ALS and FTLD-TDP brain and spinal cord tissue, along with potential phosphorylation sites [11–13, 52, 75–77]. Sites are listed by amino acid (serine (S), threonine (T), or tyrosine (Y)) and coloured by their localisation (N-terminus, purple; binding region, blue; C-terminus, orange). **C**-**F** “front”, (**G**-**J**) “left side”, (**K**-**N**) “right side”, and (**O**-**R**) “back” view of TDP-43 (Q13148) structure as predicted by AlphaFold3 and visualised in PyMOL. TDP-43 is represented in cartoon (**C**,**G**,**K**,**O**) or surface (**E**,**I**,**M**,**Q**) form, with the N-terminus in purple, binding region in blue, and the C-terminus in orange. **D**,**H**,**L**,**P** TDP-43 cartoon coloured by predicted Local Distance Difference Test (pLDDT), which shows regions with very high (dark blue, pLDDT > 90), high (light blue, pLDDT = 70–90), low (yellow, pLDDT = 50–70), and very low (orange, pLDDT < 50) confidence in the predicted structure as calculated by AlphaFold3. **F**,**J**,**N**,**R** Electrostatic surface potential (ESP) was calculated using APBS Electrostatics Plugin in Pymol where regions coloured red indicates negative potential, while regions indicate neutral potential, and blue indicate positive potential

TDP-43 is comprised of 414 amino acids that contain a NLS, two RNA-recognition motifs (RRM1, RRM2), and an intrinsically disordered C-terminal domain (CTD) consisting of glycine-rich and glutamine-asparagine-rich regions [66] (Fig. 1A). The C-terminus lacks a well-defined native structure, as highlighted by its low predicted Local Distance Difference Test (pLDDT) score, a measure of confidence in protein structure predictions, by AlphaFold3, a protein structure prediction tool [67] (Fig. 1D,H,L,P). This low score reflects the structural flexibility of the C-terminus, which enhances its mobility and malleability and allows interaction with a diverse range of molecular partners [68]. The C-terminus is also referred to as a prion-like domain due to its high proclivity for aggregation and the location of many sporadic and familial ALS/FTLD-TDP-associated mutations [69–71]. The N-terminus has been implicated in multiple roles, including its ability to promote TDP-43 oligomerization and DNA binding affinity in vitro and in vivo systems [72–74].

#### Pathology

**Fig. 2:**
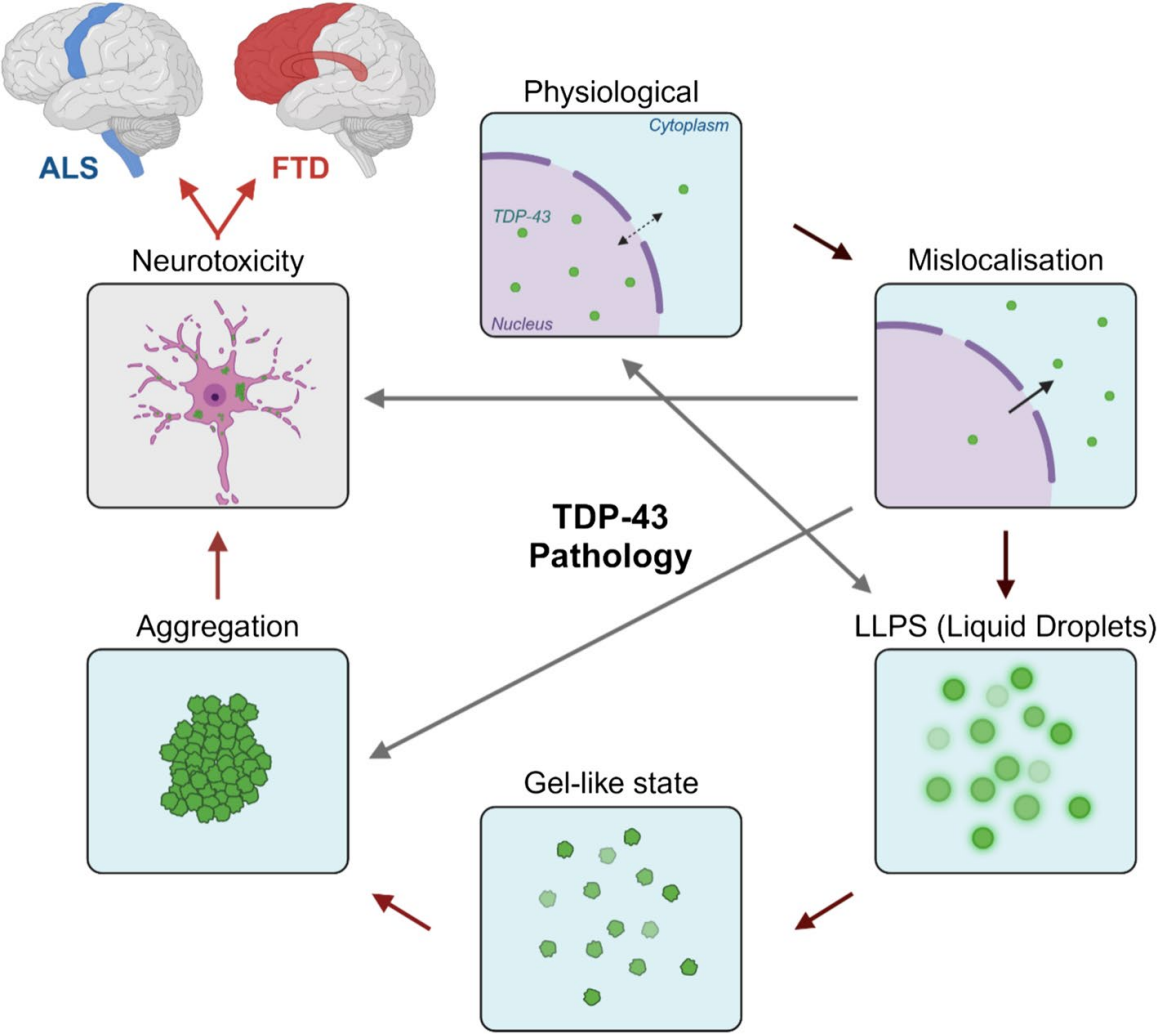
TDP-43 pathology in ALS and FTLD-TDP. Physiological TDP-43 is primarily nuclear with some cytoplasm shuttling and can undergo liquid liquid phase separation (LLPS) to form liquid droplets, membrane-less organelles that provide microenvironments for cellular processes. In disease, TDP-43 mislocalises to the cytoplasm and forms insoluble aggregates, possibly through transition from liquid droplets into less-dynamic gel-like state, then solid state aggregates. This process is toxic to the neurons due to a loss of nuclear TDP-43 and gain of TDP-43 aggregates. The neurotoxicity leads to a loss of neurons including upper and lower motor neurons within the motor cortex and spinal cord for ALS (blue), and von Economo neurons and fork cells in the frontoinsular and anterior cingulate cortices for FTLD-TDP (red). Figure constructed using biorender.com

TDP-43 pathology is a progressive process that results in the accumulation of cytoplasmic TDP-43 aggregates. This occurs notably in the upper and lower motor neurons in the motor cortex and spinal cord for ALS, and von Economo neurons and fork cells in the frontoinsular and anterior cingulate cortices in most cases of frontotemporal lobar degeneration with TDP-43 pathology (FTLD-TDP), the pathological entity which causes approximately half of all FTD [78–81] (Fig. 2). An important avenue for future research is mapping the brain regions where specific TDP-43 phosphorylation sites are detected, as selective neuronal vulnerability may be influenced by region-specific phosphorylation patterns. In disease, TDP-43 mislocalises to the cytoplasm, leading to an accumulation of cytoplasmic and loss of nuclear TDP-43 [55, 82]. The mechanisms underlying TDP-43 aggregation are complex, with emerging evidence implicating liquid–liquid phase separation (LLPS) as an intermediate phase in the transition from soluble to aggregated TDP-43 [83–90]. LLPS is the formation of membrane-less protein organelles, also known as liquid droplets, to compartmentalise various biological processes such as the spatiotemporal organisation of RNA processing [91–93]. Recent studies have revealed that TDP-43 undergoes LLPS, forming dynamic and reversible liquid-like assemblies to create specialised cellular microenvironments for RNA processing and metabolism, such as stress granules and paraspeckles [94, 95]. Additionally, TDP-43 can form spherical shells called anisosomes when unable to bind RNA through disease-related mutations or acetylation [89, 96]. Intrinsically disordered regions (IDRs), like the TDP-43 CTD, are a common feature of proteins that undergo LLPS and have been reported to mediate the dynamics of the liquid droplet [97, 98]. LLPS-mediated aggregation has been observed in other neurodegenerative proteins, including α-synuclein, tau, FUS and hnRNPA1 [93–95, 98–104]. When LLPS dynamics are disrupted, TDP-43 liquid droplets can mature into a less dynamic gel-like state before solidifying to form aggregates [36, 83]. These aggregates represent a hallmark of TDP-43 pathology and are observed in post-mortem tissue [11, 55]. Aggregated TDP-43 exhibits several PTMs including acetylation, phosphorylation, SUMOylation, and ubiquitination [11, 51, 55, 82, 105]. Several studies suggest that TDP-43 aggregation and LLPS are driven by its C-terminal IDR, the region which experiences the most pathological phosphorylation [99, 106, 107] (Fig. 1B). Phosphorylated TDP-43 aggregates have also been detected in lysosomes in post-mortem ALS/FTLD pre-frontal cortex tissue, suggesting autophagy plays a role in cytoplasmic TDP-43 accumulation [108].

TDP-43 pathology is hypothesised to drive dual toxicity: a gain-of-function toxicity through the presence of cytoplasmic aggregates and a loss-of-function toxicity due to the depletion of functional nuclear TDP-43 [36, 109–113]. Loss of functional TDP-43 affects various RNA and protein targets, leading to several detrimental events linked with neurodegenerative processes. One notable example is the mis-splicing of *UNC13A* mRNA due to TDP-43 cytoplasmic mislocalisation, causing reduced UNC13A protein expression, which is implicated in the pathogenesis of ALS and FTLD-TDP [40, 43]. Furthermore, TDP-43 pathology and phosphorylation may be controlled by an unidentified direct or an indirect mechanism, for example the accumulation of TMEM106B, a risk factor for FTD, correlates with insoluble phosphorylated TDP-43 levels in FTLD-TDP type A post-mortem tissue [114]. This evolving understanding of TDP-43 pathology offers valuable insights into the complex interplay of factors, including phosphorylation, which contribute to the pathogenesis of neurodegenerative diseases.

#### TDP-43 phosphorylation

Phosphorylation is a fundamental and reversible PTM involving the covalent attachment of a negatively charged phosphoryl group to specific amino acids, primarily serine, tyrosine, or threonine in eukaryotes [115, 116]. Structural studies suggest that serine and threonine phosphorylation have distinct conformational effects, with threonine phosphorylation inducing greater rigidity through a process known as pseudocyclization, wherein noncovalent interactions stabilize a constrained backbone conformation that mimics proline’s backbone cyclization [117]. Phosphorylated TDP-43 is a major pathological hallmark, consistently observed in ALS and FTLD-TDP but poorly detected in physiological conditions [11–13]. This suggests that TDP-43 in healthy tissue is either not phosphorylated or experiences low levels of transient phosphorylation due to an equal balance between kinase and phosphatase activity. Notably, phosphorylation at S409/410 is commonly used to identify TDP-43 inclusions in brain and spinal cord as it is highly consistent in disease and due to the development of highly specific and reliable antibodies [11–13]. Of the 64 potential TDP-43 phosphorylation sites, 27 have been detected in ALS or FTLD-TDP via mass spectrometry approaches, with six major pathological sites recognised: S369, S379, S403/404, and S409/410 [11–13, 52, 75–77] (Fig. 1B). Nineteen of these pathological sites are within the C-terminus, potentially owing to the susceptibility of IDRs to PTMs and its role in mediating LLPS [118–121]. The presence of phosphorylated TDP-43 in disease therefore raises the question of whether it contributes to disease progression and neuron loss (i.e., causative) or a cellular defence mechanism (i.e., protective).

#### Techniques for studying TDP-43 phosphorylation

To investigate the role of TDP-43 phosphorylation in disease, several in vitro and in vivo models and various experimental approaches have been utilised (Table 1). Manipulating the abundance levels of kinases and phosphatases through overexpression, knockdown/out, or pharmacological inhibition have played a prominent role in studying TDP-43 phosphorylation. Overexpression of kinases that target TDP-43 can induce TDP-43 phosphorylation in vitro and in vivo, for example TTBK1 expression in HEK293 cells increased TDP-43 mislocalisation and in *C. elegans* enhanced TDP-43 cytoplasmic accumulation and negatively impacted locomotion [22, 25]. Conversely, knockdown or knockout approaches, achieved through techniques such as RNA interference or CRISPR/Cas9 gene editing, can assist in identifying the consequences of decreased TDP-43 phosphorylation by targeting specific kinases. For instance, TTBK1 knockdown has been shown to ameliorate neurite length and neuron loss associated with TDP-43 overexpression in iPSC-derived neurons [25]. Similar to kinase manipulation, phosphatase expression can be adjusted to modulate TDP-43 dephosphorylation activity. For instance, knockout of the phosphatase calcineurin led to increased TDP-43 phosphorylation, enhanced TDP-43 accumulation, and exacerbated motor phenotypes in *C. elegans* [122].

**Table 1:** Techniques to study TDP-43 phosphorylation

Technique	Description	Outcome	Strengths	Limitations	TDP-43 studies
**Kinase overexpression**	*Increased expression of kinases*	*↑ substrate phosphorylation*	• Biologically relevant (increased kinase expression in ALS/FTD) • True phosphorylation • Reversible	• Off-target effects (enhanced phosphorylation of other substrates)	In vitro [15, 16, 18, 25, 28, 29, 31, 32, 34, 35, 56] In vivo [14, 16, 22]
**Kinase inhibition or knockout/down**	*Eliminate or reduce expression (knockout/down) or activity (therapeutic inhibition) of kinase*	*↓ substrate phosphorylation*	• True phosphorylation	• Off-target effects (reduced phosphorylation of other substrates)	In vitro [14, 15, 19, 23–28, 30, 56, 123, 173, 279] In vivo [15, 14, 17, 24, 26, 30]
**Phosphatase inhibition or knockout/down**	*Eliminate or reduce expression (knockout/down) or activity (therapeutic inhibition) of kinase*	*↑ substrate phosphorylation* *(↓ dephosphorylation)*	• True phosphorylation • Biologically relevant (decreased phosphatase expression in ALS/FTD)	• Off-target effects (enhanced phosphorylation of other substrates)	In vitro In vivo[122]
**Phosphomimicry**	*Amino acid substitution of substrate – replace phosphorylation site (serine/threonine/tyrosine) with aspartate or glutamate to mimic or alanine to prevent phosphorylation*	*Mimic or prevent phosphorylation*	• Site specific • Substrate specific	• Irreversible • Alanine loss of hydrogen bond capacity • May not be sufficient replacement for phosphorylation [126, 127, 280] - Charge difference - Smaller steric hindrance	In vitro [21, 29, 32–35, 128] In vivo [31]
**In vitro kinase assay**	*Incubation of recombinant/purified substrate and kinase or phosphatase*	*Study kinase/phosphatase and substrate interaction*	• Purified sample (for direct interaction studies)	• Artificial conditions	[14, 16, 25, 26, 29, 30, 34, 128]
**Inducement**	*Physical changes e.g. heat shock*	*↑ or ↓ phosphorylation*		• Off-target effects	[32]
**Inducement**	*Small molecule/chemical e.g. ethacrynic acid, sodium arsenite*	*↑ or ↓ phosphorylation*		• Off-target effects	[15, 19, 20, 26, 30, 32, 51, 56, 122, 123, 131, 132]
**Inducement**	*Biological/genetic e.g. TDP-43 optogenetic aggregation, inducible TDP-43 oligomerisation*	*↑ or ↓ phosphorylation*		• Off-target effects	[28, 173]
**Structural experiments**	*Experimental (e.g. cryoEM) or bioinformatic (e.g. AlphaFold2) approaches to measure TDP-43 structure*	*Determine how TDP-43 phosphorylation influences its structure*	• High resolution structural data • Provides insight into molecular mechanisms through structural changes	• Poor reliability for IDRs • Difficult to capture dynamic phosphorylation	[33]
**Coarse-grain simulation**		*Determine how TDP-43 phosphorylation influences TDP-43 dynamics, stability, and interactions*	• Models large-scale dynamics • Useful to generate hypotheses	• Artificial approach • Low reliability for highly flexibly regions like IDRs • Requires experimental validation • Limited by accuracy of force fields	[34, 128]

Since physiological TDP-43 phosphorylation is not typically detectable under basal conditions, these approaches require a model system where TDP-43 phosphorylation can be induced. Such models include using ALS or FTLD-TDP immortalised cells, inducement by cellular stress or glutathione depletion, expression of mutant TDP-43, or TDP-43 overexpression [19, 23, 24, 26, 27, 30, 123]. Manipulating these enzymes may cause off-target effects by altering other cellular pathways. For example, in addition to phosphorylating TDP-43, CK2 has over 350 protein substrates, many of which are involved in spliceosome functions [124, 125]. This makes it challenging to distinguish if pathological changes from kinase or phosphatase manipulation are due primarily to TDP-43 phosphorylation or to secondary effects of other kinase target pathways. Thus, while these approaches are powerful for studying TDP-43 phosphorylation in a disease context, their findings must be validated through complementary approaches.

**Fig. 3:**
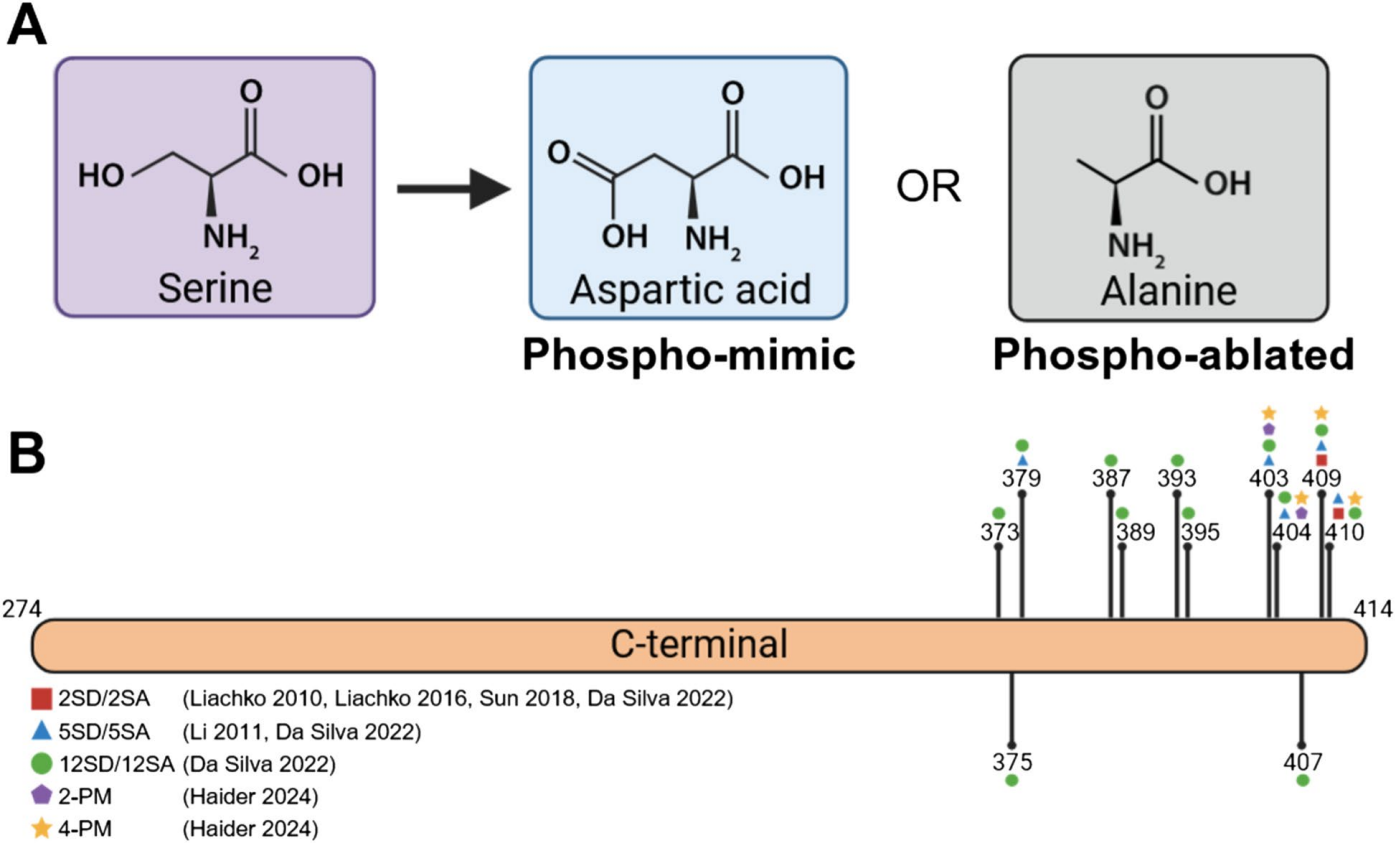
Phosphomimic TDP-43 variants used in literature. Amino acid substitution can replace serine (purple) with aspartic acid (blue) to mimic or alanine (grey) to prepare phosphorylation. **B** Phosphomimic TDP-43 variants used to study C-terminal TDP-43 phosphorylation. Figure constructed using biorender.com

Phosphomimicry is a technique to study the effects of phosphorylation at specific residues, both in vitro and in vivo. This method involves substituting phosphorylation-specific amino acids with either phospho-mimic residues, glutamic acid or aspartic acid, or phospho-ablate residues, such as alanine (Fig. 3). Glutamic acid and aspartic acid carry a negative charge that mimics phosphorylation, while alanine lacks the hydroxyl group of serine, threonine or tyrosine which is necessary for phosphorylation and therefore prevents phosphorylation. This approach allows for precise control of phosphorylation at specific residues and avoids the off-target effects of kinase overexpression. However, conflicting results raise debate as to whether glutamic acid/aspartic acid accurately represent phosphorylation as their charge and steric hinderance is reduced compared to true serine phosphorylation [34, 126, 127]. Phosphomimicry is also a permanent change unaffected by kinase or phosphatase activity, which poses a challenge for studying dynamic processes.

In vitro kinase and phosphatase assays have offered valuable insight into studying the dynamics of phosphorylated TDP-43 and its interactions with kinases and phosphatases. These assays involve the incubation of purified recombinant TDP-43 with a kinase or phosphatase and measuring the levels of phosphorylation or protein behaviour. For example, these assays have demonstrated that several kinases and phosphatases directly phosphorylate or dephosphorylated TDP-43 [11, 14, 15, 29, 35, 56, 122]. They also provide an opportunity to study TDP-43 oligomerisation and LLPS in a simplified environment that lacks regulatory elements [34]. However, this artificial environment is highly influenced by experimental conditions and lacks cellular factors which can influence protein structure, behaviour, and other PTMs. For example, Haider et al. [128] found that LLPS of CTD phosphomimic TDP-43 is dependent on salt concentration, which highlights that experimental conditions can influence findings. In vitro protein assays often do not reflect physiological levels which can lead to results that differ from in vivo conditions. Therefore, while in vitro kinase and phosphatase assays are invaluable for initial mechanistic studies, their findings must be validated in more complex in vitro and in vivo systems to ensure physiological relevance.

Additionally, several studies have reported that certain cellular stresses including impaired RNA binding (e.g. acetylation-mutant TDP-43^2 KQ^), oxidative stress (e.g. glutathione depletion), and heat shock can trigger TDP-43 phosphorylation and accumulation of insoluble TDP-43 [32, 96, 129, 130]. For example, oxidative stress from glutathione depletion caused by ethacrynic acid treatment has been reported to induce TDP-43 phosphorylation at S409/410 in HEK293T and SH-SY5Y cells [19, 26, 30, 122, 131, 132], although the relevance of this chemical stressor to disease contexts remains unclear and may be cell type dependent. These findings provide insight into how environmental factors may contribute to the pathological phosphorylation of TDP-43 and provide models to test pharmaceutical regulators [19, 32, 130].

Bioinformatic approaches may be useful to predict how TDP-43 phosphorylation influences TDP-43 structure and interactions. However, most pathological TDP-43 phosphorylation sites lie within the IDR and have low structural reliability [67, 133], such that structural tools that can predict the impact of PTMs on proteins, such as AlphaFold3, may have limited reliability in defining this region (Fig. 1D,H,L,P). This has also caused challenges for cryogenic electron microscopy and other experimental structural techniques to determine the structure of the TDP-43 CTD. Coarse-grain simulations can model large-scale dynamics and interactions, offering a broader understanding of TDP-43 behaviour influenced by phosphorylation states [34, 128]. While bioinformatic approaches are powerful tools to study many parameters in a simple and cost-effective method, currently experimental validation is vital to ensure the findings are consistent with in vivo conditions.

#### Possible interdependence of TDP-43 phosphorylation sites

A recent study of tau protein, involved in neurodegeneration, has uncovered a complex network of phosphorylation site interdependence, suggesting that certain phosphorylation sites termed ‘master sites’ influence phosphorylation at other positions within the same protein [134]. A site interdependence screen was performed in HEK293T cells expressing phosphomimic tau variants at 17 sites with or without concomitant expression of 12 different tau kinases, and phospho-epitope-specific tau antibodies targeting 10 different sites were used to detect phosphorylation. Phosphorylation at three specific threonine residues caused the most significant phosphorylation at other sites, and ablation of each of these sites decreased phosphorylation at other sites, suggesting they can control tau hyperphosphorylation [134]. Since tau and TDP-43 both undergo disease-associated hyperphosphorylation and share several kinases, TDP-43 may also have a phosphorylation interdependence network. Indeed, a recent study suggests that TDP-43 phosphorylation sites influence each other as well as the addition of other PTMs. Aikio et al. [56] found that mimicking phosphorylation at S292 independently stimulates phosphorylation at S409/410 in SH-SY5Y cells, and reduced methylation at nearby R293. To date, few TDP-43 phosphorylation sites have been characterised in this manner, largely due to the lack of commercially available TDP-43 phosphorylation antibodies across the different phosphorylation sites. Increased availability of additional phosphorylation-specific TDP-43 antibodies would greatly increase understanding of this process.

### Regulators of TDP-43 phosphorylation

**Fig. 4:**
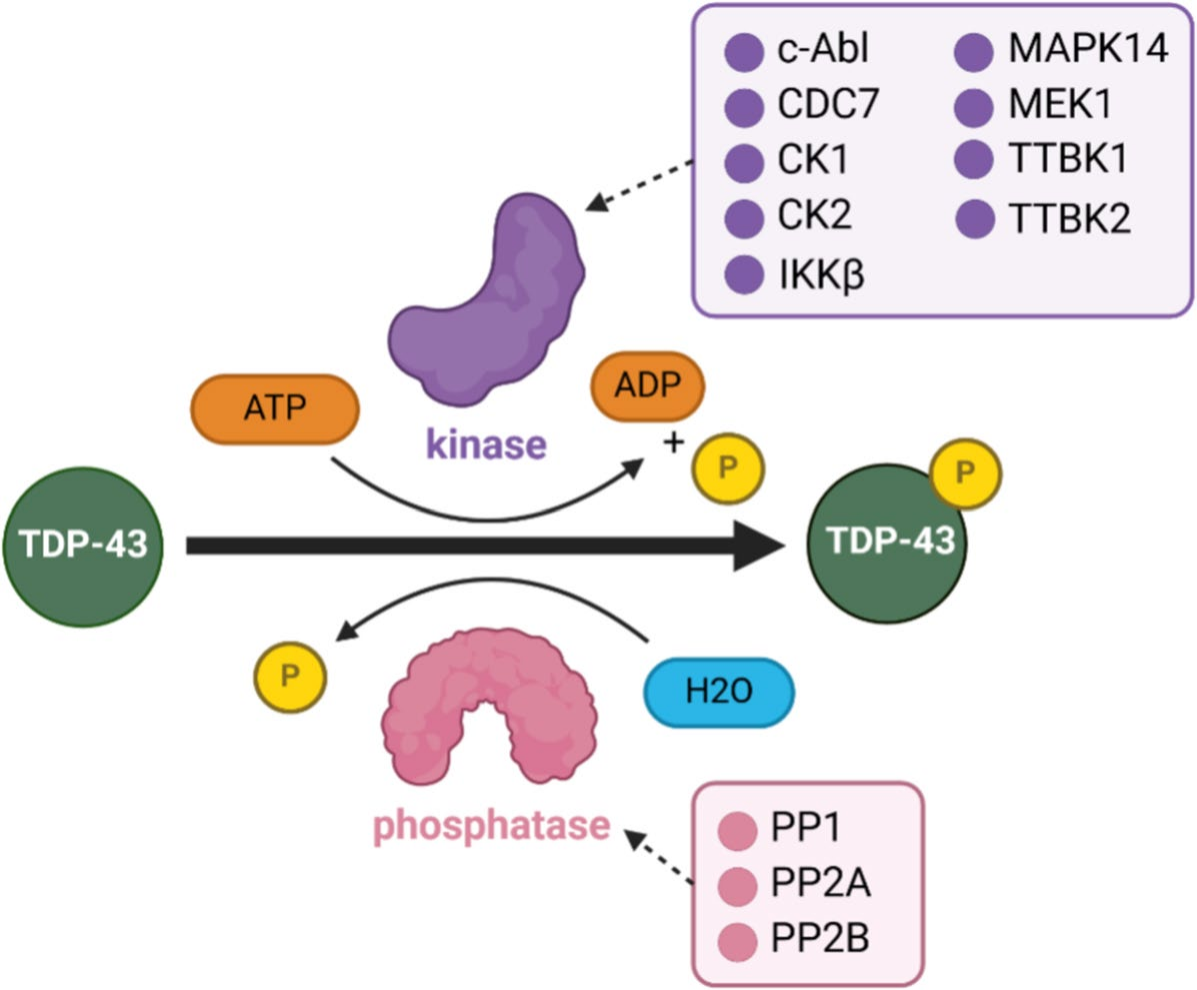
TDP-43 phosphorylation is regulated by kinases and phosphatases. Phosphorylation involves the transfer of the γ-ATP phosphate of ATP to TDP-43 by a kinase (purple). Dephosphorylation is the removal of this phosphate group by a phosphatase (pink). Reported TDP-43 kinases include c-Abl, CDC7, CK1, CK2, IKKβ, p38α/MAPK14, MEK1, TTBK1, and TTBK2 while phosphatases include PP1, PP2A and PP2B. Figure constructed using biorender.com

Phosphorylation is regulated by kinases and phosphatases, which add and remove phosphate groups from other proteins. Dysfunction in these enzymes occurs in several neurodegenerative disorders and correlates with the increased phosphorylation of aggregation-prone proteins, including α-synuclein, FUS, tau, and TDP-43 [25, 135–141]. For example, the upregulation of casein kinase 1 (CK1) in ALS, FTLD-TDP, and Alzheimer’s disease suggests that enhanced kinase activity may drive the increased phosphorylation of aggregating protein substrates including TDP-43, tau, and α-synuclein [20, 24, 142–144]. At least 9 different kinases have been reported to phosphorylate TDP-43, including c-Abl [29], Cell Division Cycle 7 (CDC7) [14], Casein Kinase 1 (CK1), Casein Kinase 2 (CK2) [11], Inhibitor of nuclear factor kappa-B kinase subunit beta (IKKβ) [35], Mitogen-Activated Protein Kinase 14 (p38α/MAPK14), Mitogen-Activated Protein Kinase Kinase 1 (MEK1) [32], and Tau Tubulin Kinases 1 and 2 (TTBK1/TTBK2) [15], with varying levels of evidence available (Fig. 4, Table 2). To date, no protein-based screen has been performed to identify the full suite of TDP-43 kinases, and whether a single or multiple kinases are most important for driving TDP-43 phosphorylation and how the kinases may influence each other remains largely unexplored.

**Table 2:** Reported TDP-43 kinases

Kinase	Description	Accession	pTDP-43 sites	In vitro assay	TDP-43 studies	Other neurodegeneration substrates	Expression and activity in primary TDP-43 proteinopathies	Expression and activity in other neurodegenerative diseases
**c-Abl**	Tyrosine-protein kinase c-Abl	P00519	Y43 [29]	[29]	[29]	α-synuclein, PD [281, 282] Parkin, PD [283] Tau, AD [284–286]	• ↑ *ABL1* RNA in post-mortem sALS spinal motor neurons [149] • ↑ c-Abl protein abundance in rNLS8 TDP-43 mouse model [143]	
**CDC7**	Cell Division Cycle 7-related protein kinase	O00311	S409, S410 [14, 26]	[14, 15]	[14, 173]			
**CK1**	Casein Kinase 1	P48729 (CK1α) P48730 (CK1δ) P49674 (CK1ε) Q9HCP0 (CK1γ1) P78368 (CK1γ2) Q9Y6M4 (CK1γ3)	S2, Y4, T25, T88, S91, S92, T116, S183, S242, S254, S273, S292, S305, S342, S347, S350, S369, S375, S377, S379, S387, S389, S393, S395, S403, S404, S407, S409, S410 [11, 167]	[11, 34, 167, 173]	[16, 18, 19, 24, 171, 173, 279, 287]	APP-β, AD [168, 288] α-synuclein, PD [171, 172] Tau, AD [169–171]	• ↑ *CSNK1D* mRNA in post-mortem sALS spinal cord and frontal cortex [24] • ↑ *CSNK1E* mRNA levels correlated with pTDP-43 in post-mortem sALS patient tissue [20] • ↑ CK1δ protein abundance in rNLS8 TDP-43 mouse model [143]	• ↑ > 30-fold CK1δ in post-mortem AD hippocampus [144] • ↑ *CSNK1D* mRNA in post-mortem AD hippocampus, amygdala, entorhinal cortex and midtemporal gyrus [142]
**CK2**	Casein Kinase 2	P68400 (CK2α) P19784 (CK2α’) P67860 (CK2β)	S379, S409, S410 [11]	[11]	[188]	APP-β, AD [168] α-synuclein, PD [172, 178–181] Tau, AD [182]		• ↓ CK2 activity in AD [289] • ↑ CK2 in post-mortem AD hippocampus and temporal cortex [290]
**IKKβ**	Inhibitor of nuclear factor kappa-B kinase subunit beta	O14920	T8, S92, S180 [35]	[35]	[35]	TDP-43, ALS, FTD [35]	• ↑ abundance in post-mortem FTLD-TDP frontal and temporal cortex [193]	
**MAPK14**	Mitogen-Activated Protein Kinase 14	Q16539	S292, S409, S410 [56]	[56]	[32]	Tau [291] TDP-43 [56]		
**MEK1**	Mitogen-Activated Protein Kinase Kinase	Q02750	T153/Y155 [32]	N/A		Tau TDP-43 [32]		
**TTBK1**	Tau Tubulin Kinase 1	Q5TCY1	S409, S410 [15, 22]	[15, 25]	[15, 22, 25, 30, 279]	APP-B, AD α-synuclein, PD Tau, AD [191, 192] TDP-43, ALS, FTD [15, 22, 173]	• ↑ TTBK1 levels in ALS post-mortem motor cortex [25] • ↑ TTBK1 in post-mortem FTLD-TDP/FTLD-tau brains [22] • ↑ TTBK1 in rNLS8 TDP-43 mouse model [143] • ↑ *TTBK1* RNA in post-mortem FTLD-TDP-43 cerebellum [193] • TTBK1 co-localize with pTDP-43 in ALS spinal cord aggregates [15] • ↑TTBK1 in FTLD post-mortem frontal cortex [15]	
**TTBK2**	Tau Tubulin Kinase 2	Q6IQ55	S409, S410 [15]	[15]	[15, 22]	TDP-43 [14, 15]	• ↑ TTBK2 in FTLD-TDP/FTLD-tau brains [22]	

*Abbreviations:* *AD* Alzheimer’s Disease, *PD* Parkinson’s Disease, *ALS* Amyotrophic Lateral Sclerosis, *sALS* sporadic ALS

Protein Phosphatase 1 (PP1), Protein Phosphatase 2 (PP2A) and Protein Phosphatase 2B (PP2B), also known as calcineurin, have been identified as TDP-43 phosphatases, although few studies have investigated the role of these phosphatases in TDP-43 pathology (Table 3). Understanding the driving forces behind TDP-43 phosphorylation, and possible dephosphorylation, is key to understanding pathology progression.

**Table 3:** Reported TDP-43 phosphatases

Kinase	Description	Accession	pTDP-43 sites	In vitro phosphatase assay	TDP-43 studies	Other neurodegenerative substrates	Expression and activity in primary TDP-43 proteinopathies	Expression and activity in other neurodegenerative diseases
**PP1**	Protein phosphatase 1	P62136 (PPP1CA) P62140 (PPP1CB) P36873 (PPP1CC)	S379, S403, S404, S409, S410 [254]	N/A	[254]	Tau [292] TDP-43 [254]		• ↓ PP2A activity in AD brain [293, 294]
**PP2A**	Protein phosphatase 2 A	Structural subunit A P30153 (PPP2R1A) P30154 (PPP2R1B) Regulatory subunit B P63151 (PPP2R2A) Q00005 (PPP2R2B) Q9Y2 T4 (PPP2R2C) P56211 (PPP2R2D) Q06190 (PPP2R3 A) Q9Y5P8 (PPP2R3B) Q9 JK24 (PPP2R3C) Q15257 (PPP2R4) Q15172 (PPP2R5A) Q15173 (PPP2R5B) Q13362 (PPP2R5C) Q14738 (PPP2R5D) Q16537 (PPP2R5E) Catalytic subunit C P67775 (PPP2CA) P62714 (PPP2CB)		N/A		α-synuclein, PD [295, 296] Tau, AD [297–302]		• ↓ PP2A activity in AD brain [293, 294] • ↓ PP2A mRNA in AD hippocampus [303] • ↓ PP2A expression and activity in frontal and temporal cortices in AD brain [304]
**PP2B**	Protein phosphatases 2B	Q08209 (PPP3CA) P16298 (PPP3CB) P48454 (PPP3CC) P63098 (PPP3R1) Q63811 (PPP3R2)	S409, S410 [122]	[122]	[122]	Tau [298, 301, 305, 306] TDP-43 [122]	• ↓ activity in sporadic and familial ALS [260, 261] • ↓ PPP3CA and PP3R1 in FTD and rNLS8 TDP-43 mouse models [143, 193]	• ↓ PP2B activity in AD brain [293, 294, 307, 308] • ↑ PP2B activity in AD brain [309]

*Abbreviations:* *AD* Alzheimer’s Disease, *PD* Parkinson’s Disease, *ALS* Amyotrophic Lateral Sclerosis, *sALS* sporadic ALS

### Putative TDP-43 kinases

#### Tyrosine-protein kinase ABL1 (c-Abl)

c-Abl is a tyrosine kinase involved in several cellular stress pathways [145], activated by various triggers including oxidative stress, hyperglycaemia, and DNA damage, to drive a signal cascade leading to cell death through apoptosis [146–148]. Increased c-Abl expression has been found in postmortem spinal cord tissue from sporadic ALS cases [149, 150]. Inhibiting c-Abl has shown promising therapeutic effects in iPSC derived motor neurons from ALS patients with SOD1 mutations, TDP-43 mutations, or sporadic ALS, as well as in a SOD1^G93A^ transgenic ALS mouse model [150, 151]. A recent study employing an in vitro kinase assay demonstrated that c-Abl can phosphorylate TDP-43 at tyrosine 43 (Y43), and a direct interaction was supported by co-immunoprecipitation experiments in SH-SY5Y cells [29]. Mimicking phosphorylation at Y43 (Y43E) promoted TDP-43 mislocalisation and stress granule formation in SH-SY5Y cells, and TDP-43 mislocalisation, insolubility, aggregation and neuronal death in primary cortical neurons [29]. However, it should be noted that TDP-43 Y43 phosphorylation has not be detected in post-mortem ALS or FTLD-TDP tissues to date. Notably, c-Abl is the only tyrosine kinase, as opposed to serine/threonine (Ser/Thr) kinases, reported to be able to phosphorylate TDP-43, although evidence for TDP-43 tyrosine phosphorylation in human disease pathology samples remains unclear.

#### Cell division cycle 7-related protein kinase (CDC7)

CDC7 is a highly conserved Ser/Thr kinase involved in crucial cellular processes such as cell cycle regulation, DNA replication, and DNA repair [152, 153]. Although CDC7 is known for its role in the cell cycle, including regulation by the zinc-finger activator DBF4 [153–158], the function of CDC7 in non-proliferating neurons is not well understood. However, CDC7 was indicated as a TDP-43 kinase through an RNA interference kinome screen in *C. elegans* based on effects on TDP-43-associated behavioural phenotypes [14]. *In vitro k*inase assays with wildtype and mutant (M337 V) TDP-43 also showed robust phosphorylation at S409/410 by CDC7, indicating a direct interaction [14, 15]. Inhibition of CDC7 has been observed to decrease, but not eliminate, TDP-43 phosphorylation in a variety of models, including SH-SY5Y cells, ALS and FTLD-TDP immortalized lymphocytes, NSC-34 cells, TDP-43^M337V^ *C. elegans,* and TDP-43^*A315T*^ mice [14, 26, 27].

#### Casein kinase 1 (CK1)

CK1 is a family of Ser/Thr kinases of seven isoforms (α, β, δ, ϵ, γ1, γ2, and γ3) involved in many pathways, including circadian rhythm, vesicular trafficking, cell cycle progression, DNA repair, and signal transduction pathways [159–165]. CK1 α, δ, and ϵ localise to the cytoplasm and nucleus while CK1γ, due to C-terminal palmitoylation, is anchored to the plasma membrane [166]. CK1 was identified as a TDP-43 kinase through an in vitro kinase assay and has since been found to phosphorylate TDP-43 at 29 sites, including S403/404 and S409/410 [11, 167]. CK1 is also implicated in other neurodegenerative disorders, including Alzheimer’s disease and Parkinson’s disease, as it can phosphorylate APP-β [168], tau [169–171], and α-synuclein [171, 172]. Enhanced CK1 expression has also been observed in ALS, FTLD-TDP, and Alzheimer’s disease [20, 24, 142, 144]. TDP-43 itself has been shown to regulate CK1δ and CK1ε expression [20, 173], indicating a complex interplay between CK1 and TDP-43 in disease contexts. Inducible oligomerisation of TDP-43 can enhance *CSNK1D* (CK1δ gene) and *CSNK1E* (CK1ε gene) expression in SH-SY5Y cells [173]. Furthermore, enhanced UV crosslinking and immunoprecipitation (eCLIP) of the frontal cortex of sporadic ALS patients found that TDP-43 binds *CSNK1E* mRNA [20]. In addition, TDP-43 knockdown decreased *CSNK1E* but not *CSNK1D* mRNA levels in motor neuron progenitors [20], suggesting that CK1ϵ may be of particular importance in ALS. A recent study highlighted CK1δ and CK1ε as major TDP-43 kinases at S409/410 by comparing the effects of seven small molecule kinase inhibitors on TDP-43 pathology in a SH-SY5Y neuroblastoma cell model [173]. However, this study leaves open the possibility that several kinases drive pathological TDP-43 phosphorylation, since inhibition of these kinases individually decreased but did not eliminate TDP-43 phosphorylation [14, 15, 173]. A recent study explored the effects of CK1ε inhibition in a cytoplasmic TDP-43 mouse model, suggesting that therapeutic inhibition of CK1ε can reduce TDP-43 phosphorylation, lower neurofilament light chain levels, and improve survival [174].

#### Casein kinase 2 (CK2)

CK2 is a tetrameric Ser/Thr kinase known for its multifaceted roles in cellular processes, ranging from apoptosis and cell survival to RNA and protein synthesis, with over 300 substrates [124, 175]. CK2 comprises two catalytic subunits (α and/or α’) and two regulatory subunits (β), and unlike other kinases is constitutively active [124, 175–177]. CK2 was identified as a TDP-43 kinase alongside CK1 through an in vitro kinase assay with wildtype TDP-43 [11]. To date, no study has comprehensively elucidated all CK2 phosphorylation sites on TDP-43, but probing with phospho-specific antibodies has revealed phosphorylation at S379, S403/404, and S409/410 [11]. Like CK1, CK2 can also phosphorylate APP-β [168], α-synuclein [172, 178–181], and tau [182], suggesting a broad involvement in neurodegenerative diseases.

#### Inhibitor of nuclear factor kappa-B kinase subunit beta (IKKβ)

IKKβ is a catalytic subunit of IκB kinase (IKK) alongside catalytic IKKα and regulatory IKKγ subunits (reviewed in [183]), controlling inflammation and other immune responses through regulation of NF-κB. A recent study demonstrated that overexpression of IKKβ, but not IKKα or IKKγ, significantly increases NF-kB activity and promotes the proteasomal degradation of cytoplasmic TDP-43 in Neuro2a cells [35]. Using LC–MS/MS analysis, it was revealed that overexpression of IKKβ induces TDP-43 phosphorylation at threonine 8 (T8), serine 92 (S92) and serine 180 (S180), and an in vitro kinase assay demonstrated that IKKβ directly phosphorylates TDP-43 at S92 [35]. Overexpression of IKKβ also decreased TDP-43 aggregation in the hippocampus of a TDP-43^3A2S^ mouse model, induced phosphorylation at S92, and decreased neuronal damage. This study suggests that IKKβ plays a role in phosphorylating TDP-43, and also in promoting TDP-43 degradation.

#### Mitogen-activated protein protein kinase 1 (MEK1)

MEK1, also known as MAP2 K1, is a key kinase in the MAPK/ERK extracellular signalling pathway, which regulates proteome stability, proliferation, differentiation, survival, cell cycle, and apoptosis [184–186]. The potential role of MEK as a TDP-43 kinase was first suggested by Li et al*.* [32], observing that MEK inhibition prevented TDP-43 phosphorylation at T153/Y155 in response to heat shock in HEK293 and SH-SY5Y cells. Heat shock, a known cellular stressor, can induce TDP-43 phosphorylation, with emerging evidence that heat shock proteins are part of a stress-responsive protective mechanism in disease [143, 187, 188], although the direct relevance of heat shock to neurodegeneration is debatable. Interestingly, overexpression of MEK1 induced TDP-43 phosphorylation in SH-SY5Y cells even in the absence of heat shock [32]. Further, inhibition of ERK, a downstream target of MEK1, did not prevent TDP-43 phosphorylation, suggesting that the downstream MAPK/ERK pathway does not necessarily influence phosphorylation. While these findings hint at a regulatory relationship, the absence of direct evidence from in vitro kinase assays leaves a direct interaction between MEK1 and TDP-43 unexplored. Further research is required to confirm MEK1 as a TDP-43 kinase and to clarify the functional significance of T153/Y155 residue phosphorylation, which is not commonly observed in disease.

#### Mitogen-activated protein kinase 14 (p38α/MAPK14)

MAPK14, also known as p38α MAPK, is a ubiquitously expressed and highly conserved Ser/Thr kinase in the MAPK family, which plays a role in various cellular processes such as transcription, differentiation, mRNA stability, cell cycle regulation, inflammation, and stress response pathways [189]. MAPK14, along with closely related MAPK11, MAPK12, and MAPK13, is activated by proinflammatory cytokines and other environmental stresses like oxidative stress, mediated by MAPK kinase kinases (MKKs) or autophosphorylation [190]. MAPK14 was first linked to TDP-43 phosphorylation via demonstration that MAPK14 knockdown or pharmacological inhibition decreased phosphorylation of TDP-43^M337V^ at S409/410 in SH-SY5Y cells [56]. Furthermore, expression of MAPK14 with a constitutively activate mutation, but not the wildtype variant, induced TDP-43 phosphorylation and enhanced aggregation and mislocalisation in SH-SY5Y cells. This suggests that MAPK14 may require extracellular signalling or stress conditions to be activated to phosphorylate TDP-43. However, this effect could be indirect due to impaired global nucleocytoplasmic transport function by MAPK14 manipulation. Co-immunoprecipitation experiments in SH-SY5Y cells showed a direct interaction between TDP-43 and MAPK14, suggesting that MAPK14 can directly phosphorylate TDP-43 [56]. Further research is needed to clarify the potential involvement of other regulators of MAPK14 relating to TDP-43 phosphorylation.

#### Tau-tubulin kinase 1 and 2 (TTBK1, TTBK2)

TTBK1 and TTBK2 are multifunctional Ser/Thr kinases involved in various cellular processes, including microtubule dynamics and neuronal development, with their name stemming from their affinity for microtubules and characterisation as tau kinases [191, 192]. Interestingly, TTBK1 and TTBK2 are the closest evolutionary relative of CK1 and are highly homologous to each other [160]. TTBK2 is ubiquitously expressed, while TTBK1 is neuron specific [191]. TTBK1 and TTBK2 were identified as TDP-43 kinases alongside CDC7 in a RNA interference kinome screen [14]. While TTBK1 and TTBK2 did not phosphorylate TDP-43 during an in vitro kinase assay, a subsequent study demonstrated that they can phosphorylate TDP-43 under conditions of optimised magnesium concentration [15]. A recent in vitro kinase assay using a truncated active form of TTBK1 also indicated that TTBK1 can phosphorylate TDP-43 [25]. Notably, TTBK1 levels are elevated and TTBK1 co-localizes with phosphorylated TDP-43 in ALS and FTLD-TDP post-mortem tissue, and also in a cytoplasmic TDP-43 mouse model [15, 22, 25, 143, 193], and TTBK2 was also elevated in the FTLD-TDP brain [22]. Despite these findings, the specific roles of TTBK1 and TTBK2 and the precise TDP-43 phosphorylation sites targeting by these kinases remain poorly characterised.

#### Physiological pathways of putative TDP-43 kinases

**Fig. 5:**
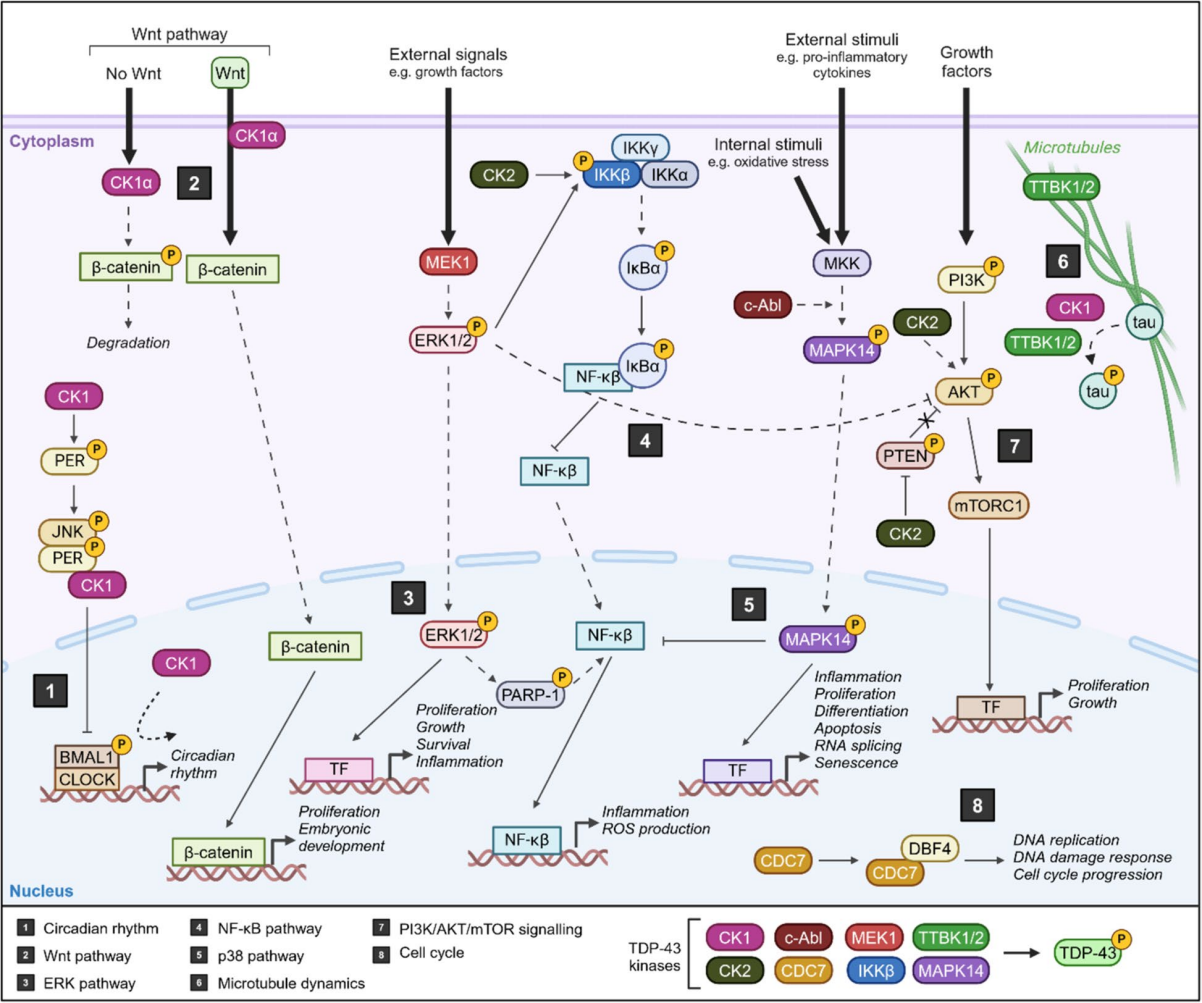
Physiological cellular pathways of putative TDP-43 kinases. Schematic of eight key biological pathways that involve c-Abl, CDC7, CK1, CK2, IKKβ, MAPK14, MEK1, TTBK1 and/or TTBK2. These pathways include 1) circadian rhythm, 2) Wnt pathway, 3) ERK pathway, 4) NF-κB pathway, 5) p38 pathway, 6), microtubule dynamics, 7) PI3K/AKT/mTOR signalling, and 8) cell cycle. Figure constructed using biorender.com

The reported TDP-43 kinases are involved in many key signalling pathways, including circadian rhythm, Wnt, ERK, NF-κB, p38, microtubule dynamics, and the cell cycle (Fig. 5). These pathways often overlap, creating a complex network that regulate cellular homeostasis, inflammation and cell division. Dysregulation of many of these pathways has been implicated in the pathogenesis of TDP-43 proteinopathies, leading to the overexpression of these kinases, which could potentially be a driving force behind aberrant TDP-43 phosphorylation. While targeting these kinases to modulate TDP-43 phosphorylation may appear to be a promising therapeutic strategy, significant challenges remain. The promiscuity of these kinases and interconnected nature of the pathways means that inhibiting one kinase could have unintended off-target downstream effects. Therefore, while kinase regulation of TDP-43 may be important in neurodegeneration, careful consideration must be given to the broader impact of therapeutic interventions targeting these kinases. Here, we will explore the known biology of the putative TDP-43 kinases focused on biological pathways that may be of relevance to consider as off-target pathways when designing therapeutic interventions to modify TDP-43 phosphorylation.

##### Circadian rhythm

CK1δ and CK1ε play key roles in circadian rhythm, which is an autonomous daily oscillation that maintains body homeostasis and plays important roles in metabolic regulation and memory consolidation. This rhythm is orchestrated by the phosphorylation of PERIOD (PER) by CK1δ and CK1ε, which leads to PER degradation and facilitates nuclear localisation, thereby modulating the length of the circadian period [194, 195]. Of note, circadian rhythm dysfunction has been identified in various neurodegenerative disorders including ALS, Alzheimer’s disease, Parkinson’s disease, Huntington’s disease, and multiple sclerosis (reviewed in [196]). In ALS SOD1^G93A^ transgenic mice, circadian rhythm dysfunction accelerated disease onset and progression through enhanced motor neuron loss, activated gliosis, and NF-κB inflammation [197]. Similar abnormalities are observed in FUS ALS mouse models, preceding cognitive impairment onset [198]. Whether these effects are related to the regulation of TDP-43 phosphorylation by CK1δ and CK1ε remains to be explored.

##### Wnt pathway

CK1α is involved in the Wnt pathway, which is integral to embryonic development and adult tissue homeostasis. It is involved in both canonical (β-catenin dependent) and non-canonical (β-catenin independent) signalling pathways, modulating various cellular processes [199–201]. Abnormal Wnt signalling has been implicated in ALS, with elevated expression of Wnt ligands, receptors, and co-receptors in ALS spinal cord tissue [202–204]. This suggests Wnt dysfunction which may contribute to disease progression.

##### ERK pathway

MEK1 kinase is involved in the extracellular signal-regulation kinase (ERK) pathway, a subset of the mitogen-activated protein kinase (MAPK) pathway, which plays a role in cell adhesion, differentiation, proliferation, and apoptosis. This pathway is activated by a series of upstream signals, including mitogens and growth factors, leading to the activation of MEK1/2 (reviewed in [205]). MEK1/2 subsequently phosphorylates and activates ERK1/2, which translocates to the nucleus where it promotes cell proliferation, growth, survival, and cytokines production. Additionally, ERK1/2 phosphorylates PARP- 1, enhancing NF-κB activity through the activation of the IκB kinase (IKK) complex. In ALS, ERK1/2 activation has been associated with disease progression, where its inhibition has been shown to provide protective effects (reviewed in [206]). ERK1/2 signalling plays a role in oligodendrocyte myelination, with emerging evidence highlighting the involvement of oligodendrocyte dysfunction in ALS [207–210]. Collectively, these findings suggest that the activation of MEK1 through the ERK pathway, through its involvement in TDP-43 phosphorylation, neuronal signalling, and oligodendrocyte function, may represent a contributing mechanism underlying ALS pathology and a potential target for therapeutic intervention. Further investigation is required into the upstream pathway of MEK1 activation and how this influences TDP-43 phosphorylation.

##### NF-κB inflammation

Putative TDP-43 kinase IKKβ is involved in the NF-κB pathway, which is one of the most significant inflammatory pathways associated with TDP-43 pathology. The canonical NF-κB pathway is activated in response to various stimuli, leading to the formation of the IKK complex, composed of catalytic subunits IKKβ, and IKKα, and the regulatory subunit IKKγ, also known as NF-κB essential modulator (NEMO) (reviewed in [211]). This complex phosphorylates Inhibitor of κB (IκB), causing IκB degradation and the release of NF-κB. The free NF-κB translocates to the nucleus to initiate the transcription of genes involved in inflammation, innate immunity, and cell survival (reviewed in [212]). Activation of NF-κB can exacerbate neurodegenerative processes by promoting neuroinflammation [213, 214], and NF-κB mRNA and protein levels are elevated in ALS patient spinal cords, suggesting activation of the NF-kB pathway [215, 216]. TDP-43 itself can regulate NF-κB pathways in both neurons and microglia [215, 217, 218]. Interestingly, neuron inhibition of NF-κB through expression of a super repressor form of IκBα in transgenic TDP-43^A315T^ or TDP-43^G348C^ mice decreased cytoplasmic TDP-43 mislocalisation, improved motor performance and cognition, and reduced motor neuron and gliosis loss [219]. Furthermore, chronic administration of LPS to activate the NF-κB pathway in TDP-43^A315T^ mice exacerbated cytoplasmic TDP-43 accumulation and aggregation [215]. These studies suggest that the NF-κB pathway worsens TDP-43 pathology and may play an important role in regulating disease pathology, potentially involving the TDP-43 kinase IKKβ. Understanding this pathway is important to more fully define the role of neuroinflammation in TDP-43 proteinopathies.

##### p38 pathway

The TDP-43 kinase MAPK14 is involved in the p38 pathway, which is another type of mitogen-activated protein kinase (MAPK) pathway that promotes inflammation, proliferation, senescence, RNA splicing, apoptosis and differentiation (reviewed in [220]). The pathway is activated by external signals including pro-inflammatory cytokines, heat shock, and UV radiation, or internal signals such as oxidative stress [221–224]. A protein cascade causes phosphorylation and activation of p38 MAPK kinases, including MAPK14, which allows entry to the nucleus. Nuclear MAPK14 inhibits NF-κB activity, promotes several transcription factors to make transcriptional changes, and the p38 pathway has been implicated in ALS pathology, particularly with SOD1 and FUS mutations (reviewed in [205]).

##### Microtubule dynamics

Putative TDP-43 kinases CK1, TTBK1, and TTBK2 play a role in microtubule dynamics [225–228]. Microtubules are intracellular structures vital for neuron development and maintenance. MTs facilitate axonal transport which is required for mitochondrial recycling, vesicle and mRNA transport, and signalling pathways [229]. Microtubules are particularly important for neurons as they play a role in neurite remodelling, generation of neuronal compartments, and growth cone mechanics [230–232]. Mutations to *TUBA4A*, a microtubules protein, cause a rare familial form of ALS, highlighting the importance of microtubules for neuron survivability. Additionally, TDP-43 interacts with microtubules for mRNP granule transport, which is vital for proper mRNA localisation and translation in neurons [59, 233]. Disruptions in microtubules dynamics can impair neuronal function and contribute to the pathogenesis of ALS, highlighting the need for further research into the mechanisms regulating MT stability and transport in neurons.

##### PI3 K/AKT/mTOR signalling pathway

The putative TDP-43 kinase CK2 is involved in the PI3 K/AKT/mTOR pathway, which is an intracellular signalling process important for cellular growth, proliferation, metabolism, and apoptosis. This pathway is activated by upstream cytokines or growth factors, such as fibroblast growth factor (FGF) and platelet-derived growth factor (PDGF), activating PI3K (reviewed in [234]). Once activated, PI3K phosphorylates and activates Protein Kinase B (AKT), leading to several downstream effects, including the activation of mammalian target of rapamycin (mTOR) [234, 235]. Activated mTOR controls macroautophagy, involved in the clearance of many cellular proteins. CK2 plays a pivotal role in this process by regulating AKT activity [236, 237]. In addition to directly phosphorylating AKT, CK2 phosphorylates the phosphatase PTEN to prevent the inhibition of AKT [236, 238, 239]. Overactivation of this pathway has been linked with several cancers, causing abnormal cell growth, proliferation, migration, and chemotherapy resistance [240, 241], suggesting that therapeutic targeting may have unintended consequences in the neurodegenerative disease context.

##### Cell cycle

Several reported TDP-43 kinases, including CDC7, CK1, MAPK14, MEK1, and IKKβ, play crucial roles in regulation of the cell cycle, particularly in response to cellular stress [228, 242]. While neurons are non-proliferating cells, evidence suggests that neurons can re-enter the cell cycle, which promotes apoptosis [243]. Aberrant neuronal cell cycle re-entry has been highlighted as a major cause of neuronal loss in Alzheimer’s disease [244–246] and ALS [247, 248]. Cell cycle-related abnormalities in ALS include hyperphosphorylation of retinoblastoma protein pRb, increased cyclin D levels, and cytoplasmic redistribution of transcription factor E2F-1 in motor neurons and glia in sporadic ALS post-mortem tissue [247]. Additionally, the cell cycle checkpoint tumor suppressor protein p53 is elevated in motor neurons in the spinal cord but not in the motor cortex in ALS postmortem tissue, further implicating cell cycle dysfuction in ALS pathology [248]. Therefore, further investigations are required to understand the role of the cell cycle in TDP-43 neurodegenerative disorders, and whether this process activates/upregulates TDP-43 kinases to regulate its phosphorylation.

### TDP-43 phosphatases

#### Protein phosphatase 1 (PP1)

PP1 is a class of multimeric Ser/Thr phosphatases responsible for a major portion of eukaryotic protein dephosphorylation [249]. This includes regulation of excitatory synaptic activity, glycogen metabolism, cell progression, cell division, apoptosis, protein synthesis, mitosis, and RNA splicing [250–252]. PP1 consists of a catalytic subunit (PPP1CA, PPP1CB, PPP1CC) and at least one regulatory subunit which confers selectivity, localisation and regulation [253]. Although the catalytic subunits have a similar sequence, the regulatory subunits are diverse and identified by their function. PP1 was found to interact with TDP-43 by co-immunoprecipitation in HEK293 cells [254]. Furthermore, overexpression of PP1α or PP1γ reduced TDP-43 phosphorylation in HEK293 cells, suggesting PP1 is important for TDP-43 dephosphorylation [254]. Notably, while Gu et al. [254] demonstrated that overexpression of wildtype TDP-43 in HEK293T cells is sufficient to detect phosphorylated TDP-43, other HEK293T [25], SH-SY5Y [18], and *Drosophila* [16] studies did not detect phosphorylation from simply overexpressing TDP-43. This difference could be due to variations in transfection protocols, such as the type of DNA delivery method or the amount of plasmid DNA used, as well as differences in stress conditions during cell culturing, which could influence the cells' response to TDP-43 overexpression. Interestingly, while PPP1 CA is downregulated in ALS/FTD frontal cortical post-mortem tissue [255], PPP1CB and PPP1CC are upregulated in FTD frontal and temporal cortex post-mortem tissue [193].

#### Protein phosphatase 2 A (PP2A)

PP2A is another class of multimeric Ser/Thr phosphatase that plays a pivotal role in regulating cellular phosphorylation events. While PP2A is ubiquitously expressed, it is most abundant in the heart and brain, with an estimate that it accounts for 71% total phosphatase activity in the human brain [140, 256]. PP2A coimmunoprecipitated with TDP-43 in HEK293 cells, suggesting a direct interaction with TDP-43 [254]. However, no in vitro assays have been performed to investigate whether PP2A is able to dephosphorylate TDP-43 directly.

#### Protein phosphatase 2B (PP2B)

PP2B, also known as calcineurin, is a conserved heterodimeric calcium/calmodulin dependent Ser/Thr phosphatase important in cellular signalling and stress responses. PP2B consists of two subunits: one of three calcineurin A (CnA) isozymes, a calmodulin-binding catalytic subunit (PPP3CA, PPP3CB, PPP3CC), and one of two calcineurin B (CnB) isozymes (PPP3R1, PPP3R2), a Ca^2+^-binding regulatory subunit [257]. PP2B plays an important role in postsynaptic structures of central synapses and synaptic endocytosis, and is activated by intracellular Ca^2+^ concentrations ([258], reviewed in [259]). An in vitro dephosphorylation assay demonstrated that PP2B can dephosphorylate TDP-43 [254]. Additionally, a yeast two-hybrid screen identified PPP3CC as a protein interactor of TDP-43^WT^, TDP-43^A315T^, and TDP-43^M337V^ [122]. In disease, PP2B has lower activity in ALS brain and spinal cord tissue [260, 261]. Downregulation of PPP3CA and PPP3R1 has been observed in post-mortem FTD-TDP temporal cortex and in the cortex of the rNLS8 cytoplasmic TDP-43 mouse model [143, 193]. Moreover, PP2B co-localises with TDP-43 aggregates in ALS and FTD post-mortem tissue, suggesting involvement in TDP-43 pathology [122].

### TDP-43 phosphorylation: pathology-driving or protective?

The literature on TDP-43 phosphorylation presents contrasting perspectives on whether it acts as a pathology-driving force, a protective mechanism, or both. The following sections will explore key aspects, including the timing of TDP-43 phosphorylation in disease progression and its roles in mislocalisation, aggregation, and neurotoxicity. Additionally, this discussion will compare studies on TDP-43 phosphorylation, highlighting major findings and models for kinase/phosphatase manipulation (Table 4) and phosphomimicry techniques (Table 5).

**Table 4:** Effects of altering kinases or phosphatase levels on TDP-43 phosphorylation in different model system

Approach	Model	Aggregation	Localisation	Neurotoxicity	Other	Reference
In vitro						
↑ kinase	SH-SY5Y	→ c-Abl overexpression ↑ cytoplasmic TDP-43 accumulation	→ c-Abl overexpression ↑ cytoplasmic TDP-43	*NR*	*NR*	Lee et al*.* 2022 [29]
↑ kinase	Cultured cortical neurons	*NR*	→ c-Abl overexpression ↑ cytoplasmic TDP-43	*NR*	*NR*	Lee et al*.* 2022 [29]
↑ kinase	HEK293 cells	→ TTBK1 overexpression ↑ insoluble TDP-43	→ TTBK1 overexpression ↑ cytoplasmic TDP-43	- TTBK1 overexpression does not impact cell death (nuclei count)	*NR*	Tian et al*.* 2021 [25]
↑ kinase	SH-SY5Y	→ Hyperactive CK1δ ↑ aggregated TDP-43 and ↓ TDP-43 solubility	→ Hyperactive CK1δ ↑ cytoplasmic TDP-43		→ Hyperactive CK1δ ↓ CFTR exon 9 skipping → Hyperactive CK1δ ↓ HDAC6 mRNA levels	Nonaka et al*.* 2016 [18]
↑ kinase	Yeast	→ Hyperactive CK1δ ↑ TDP-43 inclusions	→ Hyperactive CK1δ ↑ cytoplasmic TDP-43	→ Hyperactive CK1δ ↑ toxicity	*NR*	Nonaka et al*.* 2016 [18]
↑ kinase	Recombinant protein	← CK1δ ↑ TDP-43 solubility	*NR*	*NR*	← CK1δ ↓ phase separation	Gruijs da Silva et al*.* 2022 [34]
↑ kinase	SH-SY5Y	*NR*	→ TTBK2 overexpression → cytoplasmic phosphorylated TDP-43	*NR*	*NR*	Liachko et al*.* 2014 [15]
↑ kinase	Recombinant protein	→ CK1δ ↑ oligomerisation	*NR*	*NR*	*NR*	Hasegawa et al*.* 2008 [11]
↑ kinase	Recombinant protein	→ TTBK1 ↑ formation of high molecular TDP-43 species (rescued by TTBK1 inhibition)	*NR*	*NR*	*NR*	Tian et al*.* 2021 [25]
↑ kinase	iPSC-derived motor neurons	→ CK1ε overexpression ↑ aggregation	*NR*	*NR*	*NR*	Krach et al*.* 2018 [20]
↑ kinase	SH-SY5Y	*NR*	*NR*	→ ↑ cytotoxicity with incubation with CK1-treated oligomerised TDP-43 compared to non-treated	*NR*	Choksi et al*.* 2014 [16]
↑ kinase	HEK293T, Neuro2a	← CK2α overexpression ↓ insoluble C-terminal TDP-43 fragment (ND251, ND207)	*NR*	*NR*	*NR*	Li et al*.* 2011 [31]
↑ kinase	Neuro2a	← IKKβ overexpression ↓ aggregation	*NR*	← IKKβ overexpression ↓ toxicity of TDP-43^K181E/A321V^	*NR*	Iguchi et al*.* 2024 [35]
↓ kinase	FTLD immortalised lymphocytes	*NR*	→ CK1δ inhibition ↓ cytoplasmic and ↑ nuclear TDP-43		→ CK1δ partially reverted enhanced proliferation	Alquezar et al*.* 2016 [19]
↓ kinase	SH-SH5Y	*NR*	→ CK1δ inhibition ↓ mislocalisation	→ CK1δ inhibition ↑ cell survival from ethacrynic acid		Alquezar et al*.* 2016 [19]
↓ kinase	SH-SY5Y	→ MAPK14 inhibition ↓ aggregation of TDP-43^M337V^	→ MAPK14 inhibition ↓ mislocalisation of TDP-43^M337 V^	→ MAPK14 inhibition or knockdown ↑ survival of TDP-43^M337V^		Aikio et al*.* 2025 [56]
↓ kinase	HEK293T	*NR*	*NR*	← CK1α or CK1δ knockdown, but not TTBK1 or TTBK2, reduced TDP-43^WT^ overexpression cytotoxicity	*NR*	Deng et al*.* 2021 [28]
↓ kinase	sALS immortalised lymphoblasts		→ CK1δ inhibition ↓ cytoplasmic TDP-43			Martinez-Gonzalez et al*.* 2020 [24]
↓ kinase	N2a	→ TTBK1 knockdown ↓ insoluble TDP-43 and ↓ high molecular TDP-43 species				Tian et al*.* 2021 [25]
↓ kinase	Cultured cortical neurons from c-Abl WT or knockout mice	*NR*	*NR*	– no accumulation of insoluble TDP-43 in c-Abl KO	*NR*	Lee et al*.* 2022 [29]
↓ kinase	iPSC neurons	*NR*	*NR*	→ TTBK1 knockdown ↓ TDP-43 overexpression-induced neurite and neuron loss	*NR*	Tian et al*.* 2021 [25]
↓ kinase	NSC-34	→ CK1 inhibition ↓ aggregation induced by ER stress (tunicamycin) - CK1 inhibition does not influence TDP-43 solubility	*NR*	- *CK1 inhibition does not influence cell viability from ER stress*	*NR*	Hicks et al*.* 2020 [123]
↓ kinase	SH-SY5Y	*NR*	→ TTBK1 inhibition ↓ cytoplasmic TDP-43 induced by ethacrynic acid	→ TTBK1 inhibition ↓ cell death from ethacrynic acid	*NR*	Nozal et al*.* 2022 [30]
↓ kinase	sALS lymphoblasts	*NR*	→ TTBK1 inhibition ↓ cytoplasmic and ↑ nuclear TDP-43	*NR*	*NR*	Nozal et al*.* 2022 [30]
↓ kinase	ALS immortalised lymphocytes	*NR*	→ CK1δ inhibition ↓ cytoplasmic and ↑ nuclear TDP-43	*NR*	*NR*	Posa et al*.* 2019 [23]
↓ kinase	SH-SY5Y	*NR*	→ CDC7 inhibition ↓ mislocalisation from ethacrynic acid	→ CDC7 inhibition ↓ cell death from ethacrynic acid	*NR*	Rojas-Prats et al*.* 2021 [26]
↓ kinase	sALS lymphoblasts	*NR*	→ CDC7 inhibition ↓ cytoplasmic and ↑ nuclear TDP-43	*NR*	*NR*	Vaca et al*.* 2021 [27]
↓ kinase	FTLD-TDP lymphoblasts (GRN mutation)	*NR*	→ CDC7 inhibition ↓ cytoplasmic and ↑ nuclear TDP-43	*NR*	→ CDC7 inhibition restored CDK6 mRNA levels	Vaca et al*.* 2021 [27]
In vivo						
↑ kinase	*C. elegans*	*NR*	*NR*	→ CDC7 overexpression with M337V or WT TDP-43 ↑ neuron loss	→ CDC7 and M337V or WT TDP-43 caused paralysis and other severe effects → CDC7 overexpression is synthetic lethal with M337V but not with addition phospho-ablated mutations at S409/410	Liachko et al*.* 2013 [14]
↓ kinase	A315T TDP-43 mouse	*NR*	*NR*	→ CK1δ inhibition reduced motor neuron loss in spinal cord	→ CK1δ inhibition significantly delayed weight loss → CK1δ inhibition blocked elevated microglial cells and reduced astrocytes	Martinez-Gonzalez et al*.* 2020 [24]
↑ kinase	*C. elegans*	→ TTBK1 overexpression ↑ TDP-43 accumulation - TTBK2 overexpression does not influence TDP-43 accumulation	*NR*	- TTBK1 overexpression does not influence lifespan	→ TTBK1 overexpression ↓ locomotion - TTBK2 overexpression does not influence locomotion	Taylor et al*.* 2018 [22]
↑ kinase	*Drosophila*	← CK2α overexpression ↓ aggregation	*NR*	*NR*	*NR*	Li et al*.* 2011 [31]
↑ kinase	*Drosophila*	*NR*	*NR*	→ DBT (CK1ε homolog) enhances TDP-43^Q331K^ toxicity - DBT (CK1ε homolog) *no influence on TDP-43*^*WT*^* or TDP-43*^*M337V*^	→ DBT (CK1ε homolog) induces TDP-43 fragmentation	Choksi et al*.* 2014 [16]
↓ kinase	Recombinant protein and simulation approaches	- C-terminal phosphomimic (2PM, 4PM) TDP-43 LLPS displays biphasic dependence on salt concentration - 4PM forms smaller liquid droplets	*NR*	*NR*	*NR*	Haider et al*.* 2024 [128]
↓ kinase	TDP-43^A315T^ mice	*NR*	*NR*	→ TTBK1 inhibition ↓ motor neuron loss in ventral horn	*NR*	Nozal et al*.* 2022 [30]
↓ kinase	TDP-43^A315T^ mice	*NR*	*NR*	*NR*	→ CDC7 inhibition ↓ clasping score and ↑ time in rotarod	Rojas-Prats et al*.* 2021 [26]
↓ kinase	*C. elegans*	*NR*	*NR*	→ CDC7 null mutant or inhibition ↓ neuron loss with M337V TDP-43	→ CDC7 knockdown improved motor defects in M337V model	Liachko et al*.* 2013 [14]
↓ kinase	*Drosophila*	*NR*	*NR*	→ CK1δ inhibition ↑ lifespan		Salado et al*.* 2014 [17]
↓ kinase	*C. elegans*	*NR*	*NR*	*NR*	← Phospho-ablation at S409/410 rescued locomotion deficit, reduced paralysis, and decreased coiling from TDP-43^G290A^ or TDP-43^M337V^ expression	Liachko et al*.* 2010 [310]
↑ kinase	*Mouse*	*NR*	*NR*	← IKKβ overexpression ↓ toxicity in TDP-43 cKO model (↓ cleaved caspase 3-positive neurons)	*NR*	Iguchi et al*.* 2024 [35]
↓ phosphatase	*C. elegans*	→ PP2B knockout ↑ TDP-43 accumulation	*NR*	→ PP2B knockout exasperated loss of D-type GABAergic neurons of WT and A315T TDP-43	← PP2B knockout causes dystrophic neurites, axonal defasciculating and axon degeneration	Liachko et al*.* 2016 [122]

Symbols: protective (backward arrow), causative (forward arrow), or neutral (line)

*pTDP-43* phosphorylated TDP-43, *ICH* intracranial haemorrhage, *sALS* sporadic ALS, *NR* not reported

**Table 5:** Mimicking or preventing TDP-43 phosphorylation in different model system

Mimic/ablated sites	Model	Aggregation	Localisation	Neurotoxicity	Other	Reference
In vitro						
S48	HEK293	← S48E disrupts TDP-43 LLPS and oligomerisation	*NR*	*NR*	*NR*	Wang et al*.* 2018 [33]
379, 403, 404, 409, 410	HEK293, Neuro2a	← 5SD ↓ aggregation and ↑ solubility, 5SA ↑ aggregation and ↓ solubility of C-terminal TDP-43 fragment	*NR*	*NR*	*NR*	Li et al*.* 2011 [31]
S48	Recombinant protein	S48E disrupts TDP-43 LLPS and oligomerisation	*NR*	*NR*	*NR*	Wang et al*.* 2018 [33]
T88, S91, S92	Recombinant protein		→ Phosphomimic at T88/S91/S92 ↓ affinity for importin α1/β	*NR*	*NR*	Doll et al*.* 2022 [62]
S373, S375, S379, S387, S389, S393, S395, S403, S404, S407, S409, S410	Recombinant protein	← CK1δ and 2A, 5A, and 12A condensates have aggregate-like morphology ← 2D, 5D, and 12D condensates are more dynamic and ↓ aggregation	*NR*	*NR*	- 12D and 12 A does not impair RNA regulation	Gruijs da Silva et al*.* 2022 [34]
S373, S375, S379, S387, S389, S393, S395, S403, S404, S407, S409, S410	HeLa	← 12SD ↑solubility	- 12SD and 12SA no change in localisation or nuclear import rate	*NR*	- 12SD and 12SA does not impact TDP-43 autoregulating its own mRNA or splicing regulation	Gruijs da Silva et al*.* 2022 [34]
S373, S375, S379, S387, S389, S393, S395, S403, S404, S407, S409, S410	U2OS	*NR*	- 12SD and 12SA no change in localisation	*NR*	- 12SD and 12SA does not impact TDP-43 autoregulating its own mRNA	Gruijs da Silva et al*.* 2022 [34]
S373, S375, S379, S387, S389, S393, S395, S403, S404, S407, S409, S410	Primary neurons	← 12SD ↓ insoluble TDP-43	← 12SD ↑ TDP-43 dispersal	*NR*	- 12SD suppresses stress granule recruitment	Gruijs da Silva et al*.* 2022 [34]
T153, Y155	HeLa	← pT153/Y155 increases TDP-43 solubility from heat shock	- pTDP-43 at T153/Y155 recruited to nucleoli	*NR*	← pT153/Y155 reduces TDP-43 regulation of splicing by 30% compared to WT and T153E/Y155 A	Li et al*.* 2017[32]
T153, Y155	SH-SY5Y	- pT153/Y155 did not influence aggregation from heat shock	*NR*	*NR*	*NR*	Li et al*.* 2017[32]
Y43	SH-SY5Y	*NR*	*NR*	*NR*	← Y43E ↑ G3BP1-positive stress granules	Lee et al*.* 2022[29]
Y43	Primary cortical neurons	*NR*	→ Y43E ↑ TDP-43 cytoplasmic localisation	*NR*	*NR*	Lee et al*.* 2022[29]
Y43	Cultured cortical neurons from c-Abl WT or knockout mice	→ Y43E ↓ solubility in c-Abl WT or K/O	*NR*	→ Y43E ↑ neuronal cell death in c-Abl K/O model	*NR*	Lee et al*.* 2022[29]
S409, S410	Cultured neurons	*NR*	*NR*	→ S409/410A ↓ neuronal injury from OxyHb treatment	*NR*	Sun et al*.* 2018[21]
in vivo						
S409, S410	*C. elegans*	*NR*	*NR*	- 2SA with M337V TDP-43 and PP2B knockout did not alter neurodegeneration	- 2SA with M337V TDP-43 and PP2B knockout does not cause locomotion dysfunction	Liachko et al*.* 2016 [122]
379, 403, 404, 409, 410	*Drosophila*	← 5SE ↓ aggregates and ↑ solubility	*NR*	*NR*	*NR*	Li et al*.* 2011 [31]
S4109, 410	IHC rats	*NR*	→ S409A/S410A ↓ cytoplasmic mislocalisation	→ S409A/S410A ↓autophagy	*NR*	Sun et al*.* 2018[21]

Symbols: protective (backward arrow), causative (forward arrow), or neutral (line)

*pTDP-43* phosphorylated TDP-43, *ICH* intracranial haemorrhage, *sALS* sporadic ALS, *NR* not reported

#### Physiological TDP-43 phosphorylation

Although TDP-43 phosphorylation is strongly associated with pathological processes, some evidence suggests it may also serve a physiological role in TDP-43 function, localisation, and degradation. While phosphorylation is linked to cytoplasmic mislocalization of TDP-43 in disease, physiological TDP-43 also undergoes regulated shuttling between the nucleus and cytoplasm, indicating a potential role for phosphorylation in this dynamic process. Recent studies have identified IKKβ as a kinase capable of phosphorylating TDP-43 at residues T8, S92, S180, and S183 in HEK293T cells [35]. IKKβ overexpression reduced cytoplasmic TDP-43 and facilitated degradation of TDP-43^3A2S^ in Neuro2a cells [35]. Specifically, phosphorylation at S92 appears important for TDP-43 degradation, as the phospho-mimic S92D variant degraded significantly faster than control in Neuro2a cells, despite no changes in nuclear-cytoplasmic localization. Additionally, TDP-43 phosphorylation has been observed during cellular stress and in models expressing aggregation-prone or cytoplasm-driven exogenous TDP-43 in HEK293T cells, further supporting its role as a modulator of TDP-43 stability and stress response [96]. These findings highlight the complex interplay between TDP-43 phosphorylation, degradation, and localisation in both physiological contexts and highlights the necessity of exploring all TDP-43 phosphorylation sites.

#### Timing of phosphorylation

The timing of TDP-43 phosphorylation across the disease course is an understudied area. Emerging evidence suggests TDP-43 phosphorylation is likely not an initial mislocalisation or aggregation-inducing event but rather is triggered by ongoing pathological processes. Li et al. [31] measured a significant increase in phosphorylated TDP-43 over 48 h by expressing an aggregate-prone TDP-43 C-terminal fragment called ND251 in Neuro2a cells. Notably, this study also reported that non-phosphorylated aggregates were primarily small puncta, suggesting phosphorylation occurs after aggregation initiation and maturation. However, it is also possible that phosphorylation-specific TDP-43 antibodies have limited sensitivity, detecting phosphorylated TDP-43 only within dense aggregates. Mann et al. [262] developed a model to spatiotemporally induce TDP-43 oligomerisation in HEK293 cells by expressing TDP-43 tagged with cryptochrome 2 (CRY2), a region that undergoes reversible homo-oligomerisation when exposed to blue light. These ontogenetically induced inclusions were positive for phosphorylated TDP-43 after 4 h of continuous light, indicating that TDP-43 undergoes phosphorylation after aggregation. Another study used CRY2 optogenetics to cause multimerization of G3BP1 to induce stress granule formation in U2OS cells, showing that phosphorylated TDP-43 could be detected after 5 h of stimulated stress granule formation, demonstrating that TDP-43 recruited to stress granules becomes phosphorylated [263]. Ko et al. [173] induced TDP-43 aggregation through doxycycline-inducible expression of full length TDP-43 tagged with N50, an aggregation-inducing sequence, in SH-SY5Y and U2OS cells, inducing aggregates that were phosphorylated. Additionally, both *CSNK1D* and *CSNK1E* gene expression was upregulated in these models, suggesting cytoplasmic mislocalisation and/or aggregation triggers TDP-43 phosphorylation by upregulation of CK1δ and CK1ε. These three in vitro studies suggest that TDP-43 is phosphorylated in response to TDP-43 aggregation or stress granule recruitment [173, 262, 263]. This is supported by findings in the rNLS8 cytoplasmic TDP-43 (TDP-43^∆NLS^) doxycycline-inducible mouse model of ALS [264], in which TDP-43 phosphorylation is first detected in the cortex during early disease stages but after the accumulation of insoluble TDP-43 first begins. A study using *Drosophila* found that TDP-43 recruited to arsenite-induced or heat-induced foci were phosphorylated at S409/410 [86], but in contrast to the findings of Zhang et al. [263] where the inducement of stress granule formation through the optogenetic oligomerization of G3BP1 caused TDP-43 phosphorylation, phosphorylated TDP-43 was not detected from stress granule recruitment, highlighting how different techniques and models can produce contrasting findings. Collectively, these studies suggest that while TDP-43 phosphorylation is an early event in disease and may occur prior to disease progression, it may be a secondary event to other pathological TDP-43 features such as mislocalisation and aggregation.

#### Mislocalisation

A prevailing question revolves around whether the pathological cytoplasmic accumulation of TDP-43 results from mechanisms that actively drive TDP-43 out of the nucleus or conversely, prevents nuclear re-entry during normal shuttling. Overexpression of the putative TDP-43 kinases c-Abl, CK1δ, CK1ε, TTBK1, and TTBK2 have been reported to drive TDP-43 mislocalisation in several in vitro models [15, 18, 25, 29]. Similarly, TDP-43 mislocalisation was decreased by CK1 or TTBK1 inhibition in Alzheimer’s disease patient-derived lymphoblasts, CK1 inhibition in ALS patient-derived lymphoblasts, CDC7 inhibition in ethacrynic acid treated SH-SY5Y cells, and CDC7 inhibition in FTD and ALS patient-derived lymphoblasts, [23, 24, 26, 27]. Manipulating kinases might show effects on C-terminal phosphorylation, but phosphorylation at other sites, such as within the NLS, could influence mislocalisation and remain undetected due to the lack of suitable phosphorylation-specific antibodies.

Phosphomimicry of recombinant TDP-43 at putative phosphorylation sites (T88, S91, S92) impaired the NLS region and reduced interaction with importin α1/β, suggesting an impaired ability to re-enter the nucleus [62]. This phenomenon is observed with other aggregate-prone proteins including FUS, of which phosphorylation triggered by DNA damage hinders binding to transportin 1 (TRN1), leading to cytoplasmic accumulation [265]. Gruijs da Silva [34] found that phosphomimic substitutions at 12 C-terminal sites did not affect TDP-43 localisation or nuclear import rate in HeLa cells. These studies suggest that phosphorylation within the NLS may play a more important role than C-terminal sites in influencing cytoplasmic accumulation of TDP-43. TDP-43 phosphorylation has also been implicated in re-localisation to other subcellular compartments, including phosphorylation at T153/Y155 which induces nucleoli recruitment, and phosphomimicry (G298D) at disease-associated mutation G298S which increased TDP-43 mitochondrial localisation [32, 63]. Overall, these studies suggest that TDP-43 phosphorylation impairs nuclear entry, driving mislocalisation to the cytoplasm, although this is not consistent between models and techniques. Intriguingly, this process may serve a protective purpose by sequestering misfolded or abnormal TDP-43 within the cytoplasm where it can undergo clearance mechanisms. Further studies investigating the subcellular localisation and timing of this phosphorylation in TDP-43 pathology are required to help understand whether TDP-43 phosphorylation is a protective mechanism or drives pathology.

#### LLPS and aggregation

Accumulating evidence suggests that TDP-43 phosphorylation plays a pivotal role in LLPS and aggregate formation. A recent investigation into the role of C-terminal phosphorylation on TDP-43 phase separation dynamics tested recombinant C-terminal phosphomimic variants with varying concentrations of NaCl [128]. The salt concentration plays a role is regulating recombinant protein stability, crystallization, and behaviour by altering the ionic strength of the solvent, where higher salt concentrations support protein stability. This study revealed that phosphorylation increases LLPS in the absence of NaCl and displays a diphasic dependence on salt concentrations wherein phosphorylation decreases LLPS at higher concentrations. Gruijs da Silva et al. [34] mimicked phosphorylation at 2, 5 or 12 pathological sites (Fig. 3) and found phosphorylation reduced LLPS and aggregation to generate more liquid-like and dynamic condensates. Both studies proposed mechanisms based on coarse-grained modelling, with Haider et al. [128] concluding that the electrostatic change of phosphorylation modulates the intermolecular hydrophobic interactions that drive LLPS and Gruijs da Silva et al. [34] suggesting phosphorylation forms more protein-solvent interactions instead of protein–protein interactions.

Several studies have concluded that TDP-43 phosphorylation enhances the propensity of TDP-43 to aggregate. Phosphorylated TDP-43 induced by CK1δ or CK1ε overexpression correlated with TDP-43 aggregation in an in vitro kinase assay [11], in SH-SY5Y cells [18], and in iPSC-derived motor neurons [20]. TTBK1 overexpression decreased TDP-43 solubility in HEK293 cells, suggesting increased aggregation [25]. Additionally, CK1δ inhibition in ER-stressed NSC- 34 cells decreased TDP-43 phosphorylation and aggregation [123]. TTBK1 knockdown or inhibition decreased the abundance of high molecular TDP-43 species in Neuro2a cells [25]. Further, MAPK14 inhibition decreased aggregation in SH-SY5Y cells [56]. Inhibition of TDP-43 phosphatase PP2B increased phosphorylated TDP-43 and aggregation in HEK293 cells [122]. Similarly, PP2B knockout in *C. elegans* displayed increased TDP-43 aggregation and worse motor control phenotypes [122]. Overall, these studies suggest that TDP-43 phosphorylation exacerbates TDP-43 aggregation, which can be mitigated by decreasing phosphorylation. However, none of these studies show a direct link between phosphorylation and aggregation, since the observed outcomes could potentially be influenced by off-target effects of kinase/phosphatase manipulation.

Conversely, other studies suggest TDP-43 phosphorylation can decrease aggregation. Mimicking phosphorylation at S409/410 (2SD) reduced aggregation and enhanced solubility compared to wildtype TDP-43 in HEK293 cells [266]. Similarity, 5SD (S379/S403/S404/S409/S410D) reduced aggregation and enhanced solubility in TDP-43 C-terminal fragment HEK293T, Neuro2a, and *Drosophila* models and in in vitro aggregation assay [31, 34]. Preventing phosphorylation at these sites (5SA) enhanced aggregation in Neuro2a cells [31]. Additional C-terminal phosphomimicry (12SD, S373/S375/S379/S387/S389/S393/S395/S403/S404/S407/S409/S410D) in an in vitro aggregation assay had a greater influence on enhancing solubility than 5SD, suggesting additional phosphorylation increases TDP-43’s resistance to aggregation [34]. Overexpression of CK1δ in an in vitro aggregation assay decreased aggregation and CK2α overexpression enhanced the solubility of C-terminal fragmented TDP-43 in HEK293T and Neuro2a cells [31, 34]. Phosphorylation at other sites has also been found to play a role in TDP-43 aggregation and LLPS. Overexpression of IKKβ, a recently identified putative TDP-43 kinase that phosphorylates at T8, S92, S180, and S183, decreased aggregation of wildtype and 3 A2S TDP-43, an aggregate-prone NLS and RRM1 mutant, in Neuro2a cells [35]. Phosphomimicry at S48 disrupted TDP-43 LLPS and polymeric assembly to generate more dynamic assemblies in HEK293 cells [33]. This is consistent with Wang et al. [33] where S48 phosphomimicry of N-terminal recombinant TDP-43 impaired LLPS. Aikio et al. [56] found that phosphomimicry at S292, S409/410 or both enhanced LLPS of recombinant TDP-43. The role of TDP-43 phosphorylation in aggregation and LLPS is complex and context-dependent due to conflicting findings, various models and techniques, and multiple intermediate species. This highlights the need for further research to clarify these mechanisms and their implications for neurodegenerative diseases.

#### Neurotoxicity

TDP-43 phosphorylation may contribute to neuronal toxicity – defined as mechanisms that lead to cellular dysfunction and death-potentially by influencing TDP-43 mislocalisation and aggregation, as discussed above. However, this remains a topic of debate, with conclusions varying between studies based on different kinases and experimental models. For example, while TTBK1 overexpression enhanced TDP-43 accumulation and decreased locomotion of TDP-43^WT^ overexpressing *C. elegans*, there were no changes to lifespan [22]. Tian et al. [25] found comparable results in HEK293 cells, where TTBK1 overexpression decreased TDP-43 solubility and enhanced cytoplasmic mislocalisation without influencing cell death. However, this study also reported that TTBK1 knockdown rescued neurite shortening and neuron loss in TDP-43^WT^ overexpressing iPSC neurons and extended the lifespan of TDP-43^WT^ *Drosophila*. This is consistent with findings of Nozal et al. [30] whereby TTBK1 inhibition prevented cell death induced by ethacrynic acid in SH-SY5Y cells and ameliorated motor neuron loss in the ventral horn in TDP-43^A315T^ mice.

Similar to the effects of TTBK1, CK1 overexpression also enhances, and inhibition ameliorates, TDP-43-mediated toxicity in model systems. Choksi et al. [16] found that SH-SY5Y cell death was enhanced by incubation with oligomerised TDP-43 treated by CK1. Furthermore, overexpression of the CK1ε homolog Doubletime (DBT) in *Drosophila* enhanced toxicity of TDP-43^Q331K^, but not TDP-43^WT^ or TDP-43^M337V^. Nonaka et al. [18] demonstrated that expression of a hyperactive truncated CK1δ variant in yeast increased toxicity. Another study found that inhibition of CK1δ in SH-SY5Y cells decreased toxicity from ethacrynic acid [19]. A different approach found that knockdown of CK1α or CK1δ in TDP-43 overexpressed HEK293 cells prevented cytotoxicity [28]. In a TDP-43^A315T^ mouse model, inhibiting CK1δ prevented motor neuron loss in the spinal cord in addition to delaying weight loss and decreasing neuroinflammation as demonstrated by elevated microglial cells and astrocytes [24].

Liachko et al. [14] showed that the overexpression of TDP-43^WT^ or TDP-43^M337V^ and CDC7 enhances neuron loss in *C. elegans* and causes behavioural defects. Additionally, CDC7 inhibition prevents neuron loss from TDP-43^M337V^ expression in *C. elegans*. Another study also found that CDC7 inhibition decreased cell death from TDP-43 pathology induced through ethacrynic acid in SH-SY5Y cells [26]. A further study found that overexpression of IKKβ decreased toxicity of TDP-43^K181E/A321V^ in Neuro2a cells and with TDP-43^3A2S^ AAV expression in a TDP-43 knockout mouse model [35]. In contrast, MAPK14 inhibition or knockdown enhanced the survival of SH-SY5Y cells expressing TDP-43^M337V^. Overall, the phosphorylation of TDP-43 appears to play a role in neuronal toxicity, with effects varying between different kinases and experimental models. These findings underscore the potential for kinase-targeted therapies in mitigating TDP-43-associated neurodegenerative diseases, although further research is needed to fully understand the mechanisms involved. It is also important to consider the off-target effects of kinase/phosphatase manipulation and develop strategies to modulate TDP-43 phosphorylation in a more highly specific manner.

### Hypothesised role of TDP-43 phosphorylation

We propose that TDP-43 phosphorylation serves a physiological role in some contexts in promoting TDP-43 LLPS to facilitate cellular processes that rely on phase-separated compartments. However, under pathological conditions, such as cellular stress or other unknown trigger, the upregulation or activation of TDP-43 kinases may drive dramatically increased levels of phosphorylation. Furthermore, TDP-43 itself may regulate its own phosphorylation, as suggested by its binding and regulation of *CSNK1E* mRNA [20]. In disease, we hypothesize that phosphorylated TDP-43 becomes sequestered in liquid droplet structures, which transition in an irreversible manner to a solid-like state. This solid phase likely traps TDP-43 in a phosphorylated state, shielding it from phosphatases. The phosphorylated TDP-43 detected in post-mortem tissues may thus reflect the TDP-43 post-liquid droplet state. In addition to C-terminal phosphorylation and LLPS, it is likely other TDP-43 phosphorylation sites have different functions. For example, phosphorylation around the NLS has been linked with influencing interactions with importins and thus reducing nuclear entry [62]. Furthermore, phosphorylation at S92, a site phosphorylated by IKKβ, may play a role in TDP-43 degradation [35].

These hypotheses align with similar findings in other neurodegenerative diseases, for example α-synuclein [267, 268], FUS [269, 270], and tau [271] also experience an abnormal increase in phosphorylation in disease. These proteins, like TDP-43, can undergo LLPS [101, 272–274], transition from liquid to solid phases [100, 101], and phosphorylation has been linked with liquid droplet regulation [101, 272, 273, 275, 276]. CK1 has been implicated in the phosphorylation of these proteins, including α-synuclein [172], FUS [277], tau [169, 278], in addition to TDP-43 [11, 167]. The role of CK1 in regulating phosphorylation of multiple neurodegenerative proteins suggests it may represent a shared mechanism driving pathological phase transitions. Understanding the role and regulation of TDP-43 phosphorylation could provide valuable insights into the broader mechanisms of neurodegeneration and inform therapeutic approaches applicable across multiple neurodegenerative diseases.

## Conclusion

The current literature on TDP-43 phosphorylation has developed using a variety of models and techniques, resulting in conflicting findings and interpretations that underscore the complexity of these processes in neurodegeneration. While TDP-43 phosphorylation is consistently observed in post-mortem ALS and FTLD-TDP tissue, the influence of TDP-43 phosphorylation on disease progression remains debated. Initially, phosphorylation was hypothesised to exacerbate pathology by promoting TDP-43 mislocalisation, aggregation, and cell death. However, emerging evidence suggests that phosphorylation may modulate LLPS to decrease TDP-43 aggregation and neurotoxicity, suggesting a protective role.

This review has examined the various techniques and models used to study TDP-43 phosphorylation, each with its own strengths and limitations. These methodological differences, along with the uncertainty about the timing of phosphorylation disease, complicate the ability to draw definitive conclusions. Furthermore, the possibility that TDP-43 mislocalisation and/or aggregation itself drives TDP-43 phosphorylation suggests that current approaches may not fully capture the complexity of the disease process. Given these challenges, there is a pressing need to develop new techniques and approaches to accurately assess the timing and consequences of phosphorylation at different TDP-43 sites. This includes developing a more thorough array of well-validated TDP-43 phosphorylation-specific antibodies, targeting sites such as S92 – which has been implicated in TDP-43 degradation – which will be crucial for understanding the role of pathological TDP-43 phosphorylation but may also reveal insights into the potential role of TDP-43 phosphorylation under basal conditions. The development of TDP-43 phosphorylation-specific nanobodies offers a promising method for real-time detection in live cells, helping to uncover the time and location where phosphorylated occurs. Furthermore, the impact of TDP-43 phosphorylation on interactions with other proteins remains largely unexplored. Identifying the phosphorylated TDP-43 proteome will provide valuable insights into its function consequences. Finally, identifying the full suite of TDP-43 kinases, deciphering their regulation mechanisms, and determining the specific sites they target are critical steps toward unravelling the upstream drivers of pathological phosphorylation. For example, it remains unclear whether disease-related alterations in levels of function of TDP-43 kinases has downstream effects on other target proteins which could concurrently affect disease mechanisms. Further research is crucial to understand the driving forces and consequences of TDP-43 phosphorylation and to identify therapeutic targets that can effectively regulate TDP-43 pathology while minimizing off-target effects, ultimately improving neuronal health in ALS and FTLD-TDP.

## Data Availability

Not applicable.

## Abbreviations

AD: Alzheimer’s disease
ALS: Amyotrophic lateral sclerosis
FTD: Frontotemporal dementia
HD: Huntington’s disease
IDR: Intrinsically disordered region
LCD: Low-complexity domain
LLPS: Liquid–liquid phase separation
NLS: Nuclear localation signal
PD: Parkinson’s disease
PTM: Post-translational modification
RRM: RNA-recognition motif
